# Chemotaxonomic Classification Applied to the Identification of Two Closely-Related *Citrus* TCMs Using UPLC-Q-TOF-MS-Based Metabolomics

**DOI:** 10.3390/molecules22101721

**Published:** 2017-10-13

**Authors:** Si-Yu Zhao, Zhen-Li Liu, Yi-Song Shu, Meng-Lei Wang, Dan He, Zhi-Qian Song, Hong-Lian Zeng, Zhang-Chi Ning, Cheng Lu, Ai-Ping Lu, Yuan-Yan Liu

**Affiliations:** 1 School of Chinese Materia Medica, Beijing University of Chinese Medicine, Beijing 100029, China; siyuxinxiang@126.com (S.-Y.Z.); ferove@bucm.edu.cn (Y.-S.S.); WangML1993@bucm.edu.cn (M.-L.W.); alice1993tcm@163.com (D.H.); zh_lianlian@126.com (H.-L.Z.); 2 State Key Laboratory of Bioactive Substance and Function of Natural Medicines, Institute of Materia Medica, Chinese Academy of Medical Sciences and Peking Union Medical College, Beijing 100700, China; 3 Institution of Basic Theory, China Academy of Chinese Medical Sciences, Beijing 100700, China; szy0801_2001@126.com (Z.-Q.S.); yizhangyichi1573@sina.com (Z.-C.N.); 4 Institute of Basic Research in Clinical Medicine, China Academy of Chinese Medical Sciences, Beijing 100700, China; 5 School of Chinese Medicine, Hong Kong Baptist University, Hong Kong, China

**Keywords:** chemotaxonomic classification, ultra-performance liquid chromatography-quadrupole-time-of-flight-MS (UPLC-Q-TOF-MS), metabolomics, closely-related *Citrus* TCMs, primary and secondary metabolites, metabolic pathway

## Abstract

This manuscript elaborates on the establishment of a chemotaxonomic classification strategy for closely-related *Citrus* fruits in Traditional Chinese Medicines (TCMs). UPLC-Q-TOF-MS-based metabolomics was applied to depict the variable chemotaxonomic markers and elucidate the metabolic mechanism of *Citrus* TCMs from different species and at different ripening stages. Metabolomics can capture a comprehensive analysis of small molecule metabolites and can provide a powerful approach to establish metabolic profiling, creating a bridge between genotype and phenotype. To further investigate the different metabolites in four closely-related *Citrus* TCMs, non-targeted metabolite profiling analysis was employed as an efficient technique to profile the primary and secondary metabolites. The results presented in this manuscript indicate that primary metabolites enable the discrimination of species, whereas secondary metabolites are associated with species and the ripening process. In addition, analysis of the biosynthetic pathway highlighted that the syntheses of flavone and flavone glycosides are deeply affected in *Citrus* ripening stages. Ultimately, this work might provide a feasible strategy for the authentication of *Citrus* fruits from different species and ripening stages and facilitate a better understanding of their different medicinal uses.

## 1. Introduction

Herbal medicines are widely used around the world and are gaining more attention in many fields due to their low toxicity and therapeutic performance [1,2]. Traditional Chinese Medicines (TCMs) have a long and storied history of use in China and were first documented in approximately 200 AD in the text ‘Shen nong ben cao jing’. Most herbal medicines are homologous in medicines and foods such as *Citrus*, *Dioscorea*, etc. It is well acknowledged that herbal medicines exert their curative effect via a holistic mode through primary metabolites and especially secondary metabolites [3]. The quality and content of active metabolites in herbs are highly variable, depending on factors such as the species, growth stage when harvested and geographical origins, climate and cultivation [4]. The resources and cultures of TCMs are profound and magnificent and have a long history. TCMs derived from different species, different development stages or different processing procedures exert different efficacies, which has been verified by modern pharmacology to correspond to variations in active metabolites. Therefore, the efficacy, based on variable metabolites, is an important measure to ensure the safety and effectiveness of TCMs in clinical use [5]. In the present paper, we focused on two main factors, species and development stage, to elucidate how their variation is related to the presence of primary and secondary metabolites.

DNA sequence analysis and traditional taxonomic procedures based on phenotypic traits were applied for species authentication [3]. However, only using one taxonomic method is not sufficient for quality control of TCMs. For instance, TCMs from the same species, but different development stages can hardly be discriminated by DNA sequencing [6]. Alternatively, chemotaxonomic classification provides an efficient methodology based on the diversity of plant metabolites to achieve species identification, and the method could contribute to the identification of relationships among development stages. It has been estimated that the total number of metabolites in plants is over 200,000, and each plant may contain its own chemotypic expression pattern [7]. Metabolites are the end products of the response to genetic and enzyme regulated biosynthesis. The pattern of metabolites should be more closely related to the genotype of an organism than to classic morphological traits, which are mainly based on what we can observe about the characteristics of an organism [3]. The chemotaxonomic strategy has been successfully used to reveal the functions of genes involved both in primary plant metabolism and secondary metabolism [8,9,10,11]. In addition, studies of the interactions between species and their development stage have revealed comprehensive and coordinated reprogramming of metabolic pathways via different enzyme catalysis.

Metabolomics reveals global metabolic networks, which reflect the pathophysiological states through the fluctuation of primary and secondary metabolites and can serve as a valuable tool in understanding the biosynthesis mechanisms and variable markers of TCMs [12]. Metabolomics platforms using gas chromatography-mass spectrometry (GC-MS), liquid chromatography-mass spectrometry (LC-MS) or nuclear magnetic resonance (NMR) methods have been reported to be effective tools for quality control of medicinal plants and their products [12]. Ultra-performance liquid chromatography-quadrupole-time-of-flight-MS (UPLC-Q-TOF-MS) is one of the most powerful analytical tools for the identification and discrimination of metabolic profiles in plant extracts, allowing metabolite screening to be performed over a wide range of concentrations [1,13]. The classification of global metabolites based on metabolomics is emerging as a powerful chemotaxonomic tool that provides a detailed view of the differences and similarities between herbal medicines, and it has been successfully applied for quality control of herbs, foods and their products [9]. In this paper, metabolomics using an UPLC-Q-TOF-MS method coupled with multivariate statistical analysis was applied for chemotaxonomic classification of *Citrus* fruits from different species and different development stages.

*Citrus* fruits, which are the most popular TCMs of homology of medicine and food, are of great interest because they contain large amounts of dietary phenoloids possessing important therapeutic properties for human health, and *Citrus* fruits and their juices are consumed in large quantities around the world [14]. Four different *Citrus* TCMs recorded in Chinese Pharmacopeia, 2015 edition, Zhishi (ZS, young fruit of *C. aurantium* L., harvested in May and June), Zhiqiao (ZQ, immature fruit of *C. aurantium* L., harvested in July), Qingpi (QP, pericarps of the young or immature fruit of *C. reticulate* Blanco, harvested in June) and Chenpi (CP, pericarps of the ripe fruit of *C. reticulate* Blanco, harvested in August–December), were used as research subjects [15]. Moreover, as a famous medicinal herb, *Citrus aurantium* L. is a special genus, which is composed of thin pulps and thick peels, especially in the young and immature stage. The peels contained a huge proportion of active phenolic compounds versus the full fruit. The comparison between the peels, pulps and fruits of *Citrus aurantium* L. have been put in the Supplementary Material. In the following research, the composition of peels and full fruits from *Citrus aurantium* L. was used in the same. Although they belong to the same genus, their efficacy and clinical indications are different. As we know, *Citrus* fruits contain a large number of bioactive phenolic metabolites, which have been associated with lower risks of different types of cancers and cardiovascular diseases and have been shown to exhibit antioxidant, anti-inflammatory and anti-ageing activities [14,16,17,18]. According to our previous research, the total phenolic products could be used as characteristic markers for species discrimination of different Zhishi samples, while phenolic metabolites varied over the ripening process [19]. Therefore, *Citrus* TCMs from different species (*C. aurantium* L. and *C. reticulate* Blanco) and different development stages (ZS/ZQ and QP/CP) were divided into three groups to explore their potential chemotaxonomic bioactive markers and query their corresponding biosynthesis pathways.

As a result, a metabolomics study aimed at primary and secondary metabolites from three groups of *Citrus* TCMs was performed using UPLC-Q-TOF-MS analysis. The two basic species of *C. aurantium* L. and *C. reticulate* Blanco were clearly differentiated based on their primary and secondary metabolite profiles using hierarchical cluster analysis (HCA) and orthogonal signal correction partial least squares-discriminate analysis (OPLS-DA). In addition, the variation of secondary metabolites was further used to determine the development stage, as depicted in the HCA-dendrogram and OPLS-DA score plot. Using two-stage receiver operating characteristic (ROC) analysis and the Plackett–Burman (PB) test, 79 secondary (among them, 35 metabolites were unambiguously identified compared with the reference standards) metabolites were recognized as potential chemotaxonomic markers related to biosynthetic pathways. Multivariate statistical analysis coupled with MetaboAnalyst (www.metaboanalyst.ca) permitted the comprehensive metabolomic data to be visualized and interpreted and supported integrative pathway analysis for both primary and secondary metabolites. Therefore, we propose that the chemotaxonomic classification approach focus on metabolomics-guided metabolic pathway interrogation to reveal different pharmacodynamical mechanisms of metabolites, which will be an effective strategy for quality control of closely-related TCMs and facilitate a better understanding of their different medicinal uses.

## 2. Results and Discussion

### 2.1. Optimization and Validation of the Method

#### 2.1.1. Optimization of Chromatographic Conditions

Different mobile phase compositions were screened to obtain LC chromatograms that had good peak shapes and separation. It was found that methanol and 0.1% acetic acid were the most suitable eluting solvent system. In order to acquire better sensitivity for base ions of most compounds in the TOF-MS spectra, the ionization parameters, including the desolvation gas flow rate, desolvation gas temperature, fragmentor voltage, nebulizer pressure and capillary voltage, were optimized. The optimum conditions of TOF/MS were decided as follows: capillary voltage positive ion mode 3.0 kV/negative ion mode 3.5 kV, desolvation gas flow 800.0 L/h, desolvation gas temperature 400 °C and nebulizer pressure 40 psi.

#### 2.1.2. Validation of the Analytical Method

To ensure the stability of the sequence analysis, a quality control (QC) sample was prepared by pooling a same volume (100 μL) from each *Citrus* sample and then preparing the pooled QC sample in the same way as the samples. To provide assurance that the system was suitable for use, five pooled QC samples were run prior to analysis. These pooled QC samples were also interspersed between every five samples during the analytical run.

The precision of the method was validated by the determination of the intra- and inter-day variances. For the intra-day test, the samples were analyzed six times within the same day, and for the inter-day test, the samples were examined three times per day for three consecutive days. The relative standard deviations (RSD) from UPLC-Q-TOF-MS are listed in Tables S1 and S2. To confirm the repeatability, six replicates of the QC sample were analyzed. Reproducibility was analyzed by repeating the whole method for the same sample within one day using UPLC-Q-TOF/MS (*n* = 6). For most of the metabolites investigated, the peak area RSD values of reproducibility were 0.69–2.79%.

The present method was found to have acceptable precision for the intra-day RSD values (0.36–2.92%) and inter-day RSD values (1.45–3.36%). The samples and analytical method exhibited good stability, with an RSD range of 0.04–6.31%. The method validation terms are summarized in Tables S1 and S2.

### 2.2. Identification of Primary Metabolites

As the central metabolism in plants, primary metabolism has been referenced in the assimilation, respiration, transport and differentiation processes that take place in cells [20]. It is well known that primary metabolites determine relevant crop quality traits related to nutritional content and composition, for they include a wide range of intermediate compounds and end products of metabolic pathways that accumulate in sink organs (e.g., seeds, fruits and tubers), such as lipids, amino acids and organic acids [21]. Most recently, Jing et al. [22] found that primary metabolites are fundamental to genetic improvement and metabolic engineering of either metabolic composition or plant primary production.

In the present study, following pretreatment of the metabolite data, eighty-eight metabolites were detected, including 13 glycerophosphatidic acid (GPA), 13 monogalactosyl (MG), 6 glycerophosphoethanolamine (GPEtn), 3 Glycerophosphatidylinositol (GPIns), 3 Glycerophosphatidylserine acid (GPSer), 1 Glycerophosphatidylglycerol (GPGro), 8 diacylglycerol (DG), monogalactosyldiacylglycerol (MGDG), digalactosyldiacylglycerol (DGDG) and sulfoquinovosyldiacylglycerol (SQDG), 4 phosphatidylcholine (PC), 10 phosphatidylethanolamine (PE), 5 amino acids, 3 phenolic acids, 2 nucleosides, 2 organic acids and some others. They are tentatively identified in Table 1 and Table S3. All of the primary metabolites were putatively identified according to the retention time, accurate mass, MS^2^ and searches of the Human Metabolome Database (HMDB) and LIPID MAPS Structure Database (LMSD) metabolomics databases. The LMSD is a relational database containing structures and annotations of biologically-relevant lipids [23].

Plant cells have numerous membranes that are generally the same as those in animal cells, including the plasma, mitochondrial, nuclear and peroxisomal membranes. In addition, plant cells contain unique membrane-bound compartments, the chloroplast, vacuole and symbiosome, which have other cellular structures composed of lipids, e.g., the cuticle of epidermal cells [24]. As the key photosynthetic organelle in plants, the focus of plant lipid research has been on the chloroplast being predominant; its known membrane was unique in its lipid composition, with two galactolipids, monogalactosyldiacylglycerol (MGDG) and digalactosyldiacylglycerol (DGDG) [24,25]. They also contain a significate unique sulfolipid, sulfoquinovosyl diacylglycerol (SQDG), whose head group was a modified galactose. Nevertheless, the phospholipid components of plastids are less abundant. On the other hand, the two most abundant classes of phospholipids in plant mitochondria are similar to yeast and mammals, which are phosphatidylcholine (PC) and phosphatidylethanolamine (PE), the “star products” in lipid research [26]. PC species are not only an essential component of biomembranes, but also protect cells and their organelles from oxidative stress, lipotoxicity and endoplasmic reticulum (ER) stress [26]. PE species have been reported as the second most abundant membrane phospholipid in plant cells, and they have been identified as modulators in cold temperature stress. The other productions of biologically-active lipids, glycerophosphatidic acid (GPA) and glycerophosphoethanolamine (GPEtn), are derivatives of glycerophospholipids. GPA has been reported to be the simplest phospholipid and is also one of the building units for phospholipid biosynthesis [27].

Some other primary metabolites that are detected, including amino acids, phenolic acids, nucleosides and organic acids, have been reported to be essential elements in plant cells. Amino acids are unarguably the most important metabolites from a biological point of view. Not only are they the basic structural units of proteins, but also a source of energy. Furthermore, some amino acids play important roles as neurotransmitters [28]. Now, much more attention has been paid to nucleosides, nucleotides and nucleobases, as they are the group of highly active metabolites, exhibit anti-platelet aggregation, antiarrhythmic, anti-oxidant, anti-seizure and anti-tumor effects [29]. Furthermore, phenolic acids have been found to be stronger antioxidants against free radicals and other reactive oxygen species, which are essential in preventing chronic human diseases such as cancer and cardiovascular diseases [18].

#### PCA Analysis of Primary Metabolites

**Figure 1:**
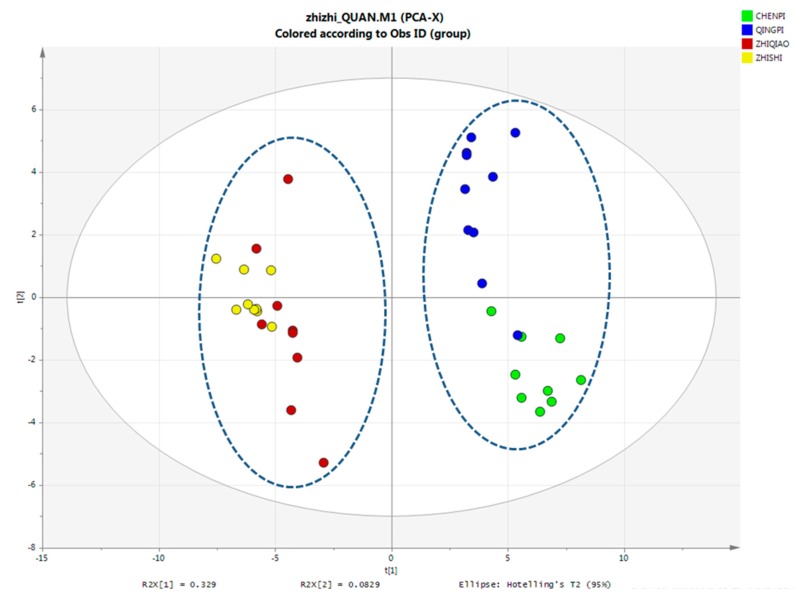
The PCA plot of primary metabolites using SIMCA (13.0 version). The data are UV scaled.

PCA was carried out to provide additional insight into the chemical differences between the *Citrus* samples. PCA is an unsupervised pattern recognition method that is used for analyzing, classifying and reducing the dimensionality of numerical datasets in multivariate problems. This approach has been widely used for quality control of herbal medicines. The contents of the 88 analytes were set as the variables, and 37 sample batches were set as the observations. The score scatter plot is shown in Figure 1, and the first two principal components accounted for 75.2% of the variance. According to the PCA score plot, all of the QP and CP samples were clustered in a small region and can be separated from the ZS and ZQ samples; the QP and CP, as well as ZS and ZQ samples from the same species might be more difficult to divide, as the collection stage may be closer. Although some inevitable differences arise from cultivation regions, harvesting time and the storage process may substantially influence the contents, and the species source was one of the most important factors. From this result, primary metabolites can be easily clustered within the different species of *Citrus*.

### 2.3. Identification of Secondary Metabolites

Compared with the advent of modern DNA sequencing techniques, chemotype-based classification methods, which are mainly based on *Citrus* secondary metabolites, are undeniably the most useful characteristic accompanying morphology in the discrimination of *Citrus* species. In current studies, it seems that a certain group of *Citrus* TCMs, which are defined by their morphology, tends to have a stable pool of secondary metabolites, which is a good indication of their classification. For instance, some articles have indicated that chemotype-based classification methods are reliable [3].

Phenolic metabolites are one of the most important groups of plant secondary metabolites; they are widespread in plants and encompass more than 8000 molecules [30]. Numerous studies have focused on polyphenols as they are a group that has multiple biological effects. Polyphenols are involved in many plant processes, such as development (participating in plant hormone signaling or pollen germination), reproduction (pigments attracting pollinators) and plant defense (protecting from UV, pathogens and predators) [31,32]. Polyphenols also play a significant part of food quality and taste, as well as potentially participating in the prevention of chronic diseases in relation to their antioxidant properties [33].

#### Non-Targeted Analysis of Secondary Metabolite Features

**Figure 2:**
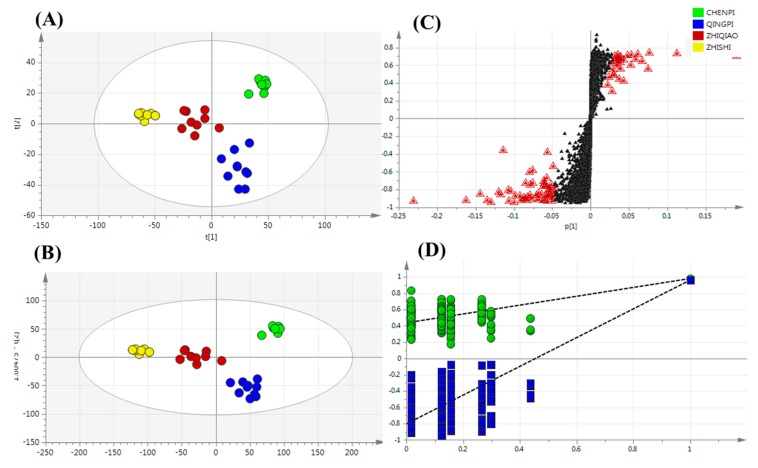
Multivariate statistical analysis of secondary metabolites: (**A**) PCA score plot; (**B**) PLS-DA score plot; (**C**) S-plot of PLS-DA (VIP > 1.0 have been marked); (**D**) cross-validation plot of the PLS-DA model with 100 permutation tests. The most differential compounds are marked with red triangles.

The plot of PCA scores clearly indicated that the first two PCs were able to separate the different developmental and ripening stages in *Citrus* (Figure 2A); 72.12% of the variance was explained using the first two PCs (scores) alone. The clear separation of the four samples of QP, CP, ZQ and ZS in the score plot indicated a more significant effect of the harvest season than variety on the *Citrus* metabolome. According to the 2015 edition of the Chinese Pharmacopoeia, ZS is harvested in May and June ZQ in July, CP in August–December and QP in June. Due to the passage of time, the quality and quantity of phenolic metabolites in ZS samples tend to be similar to those in ZQ samples. Therefore, the PCA score plot indicated that the first two components were able to separate the different species of the *Citrus* samples, as well as the different developmental and ripening stages of those *Citrus* samples, which indicated that multiple sources were important factors that can affect the use of TCMs. Of course, differences in the developmental and ripening stages should not be neglected.

A supervised PLS-DA approach was used to investigate the metabolites that showed the greatest differences. As shown in Figure 2B, the *Citrus* samples were separated into the QP, CP and ZS, ZQ samples by PC 1, and the QP samples were easily separated from the CP samples by PC 2, which indicated different secondary metabolite phenotypes for *Citrus* picked at different ripening stages. Cross-validation analysis showed that the R^2^ and Q^2^ intercepts were 0.487 and 0.894, respectively, thus demonstrating that the PLS-DA model was reliable (Figure 2D). An S-plot was used to discover key metabolites that contributed to the differentiation of the QP, CP, ZQ and ZS samples (Figure 2C). Seventy-nine metabolites, including 14 flavanones, 24 flavone and flavone glycosides, 4 flavonol and flavonol glycosides, 29 coumarins, 1 anthocyanin, 4 limonoids and glycosides and 1 abscisic acid, were tentatively identified (Table 2). The secondary metabolites were putatively identified according to the retention time, accurate mass, MS^2^ and searches of the PubChem and ChemSpider databases. ChemSpider (http://www.chemspider.com/) is a free chemical structure database providing fast access to over 34 million structures, properties and associated information. Among them, 35 metabolites were confirmed by authentic standards. Others were putatively identified according to retention time, accurate mass, MS^2^ and metabolomics databases.

**Figure 3:**
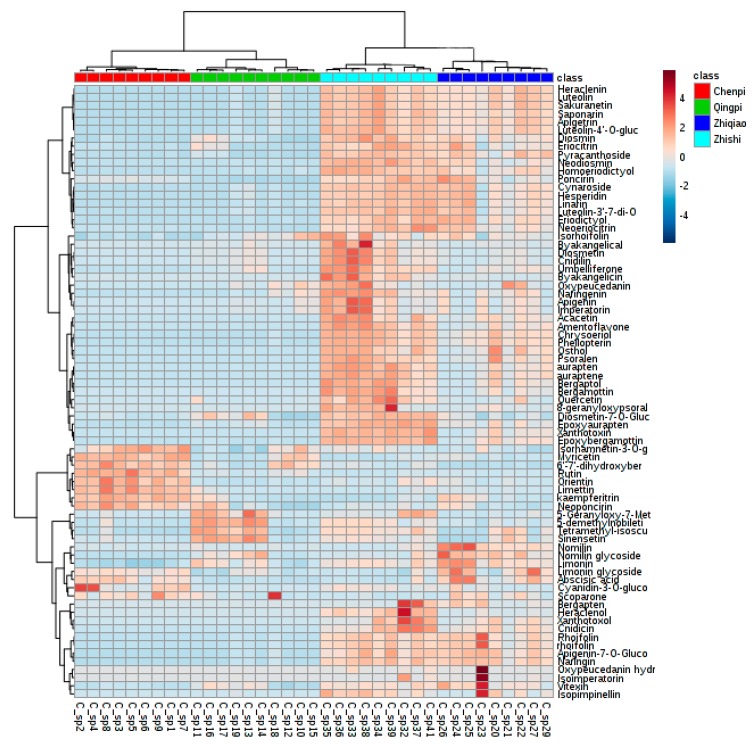
Heat-map of metabolite contents in Qingpi, Chenpi, Zhishi and Zhiqiao. The data are UV scaled.

A heat-map was applied to visualize the developmental variations of differential metabolites in the *Citrus* samples (Figure 3). The data were Pareto scaled. A red box indicates that a metabolite occurred at greater than the mean level in a sample, and a blue box means that the metabolite was at a lower level. Flavonol and flavonol glycosides, some coumarins (6′-7′-dihydroxybergamottin and psoralen) and anthocyanin clearly occurred at higher levels in the QP and CP than in the ZS and ZQ samples; while others, flavone and flavone glycosides, flavanones, majority coumarins and limonoids and glycosides, were found to be significantly higher in ZS and, especially, in ZQ samples. Otherwise, some coumarins (bergapten, xanthotoxin, isopimpinellin, aurapten, auraptene and bergaptol) and some flavone and flavone glycosides (amentoflavone and acacetin) were at their lowest levels in ZQ samples.

### 2.4. Variable Selection and Annotation of Metabolites

#### 2.4.1. Chemotaxonomy of Citrus by Primary and Secondary Metabolites

The databases we obtained were introduced to the HCA technique, taking into account the concentration of each sample. HCA examines the distances between the samples in a dataset, and the information is represented in a two-dimensional plot (dendrogram). The most similar points were grouped and then formed clusters [34]. The process is repeated until all of the points are inserted into a unique group. The style of the data is auto-scaled, and the Euclidean distance with the complete linkage method is used to calculate the sample similarities. A hierarchical agglomerative procedure is employed to establish clusters.

**Figure 4:**
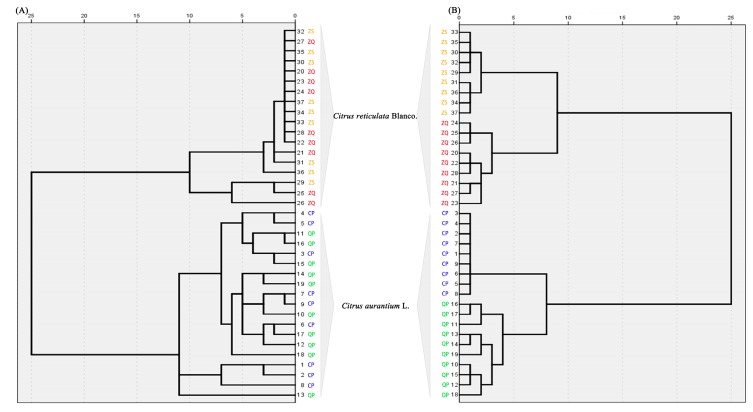
Hierarchical cluster analysis (HCA) of *Citrus* samples. (**A**) The dendrogram of primary metabolites between the production of *Citrus reticulate* Blanco and *Citrus aurantium* L. in the species; (**B**) the dendrogram of secondary metabolites between *Citrus* samples.

The results showed groupings of primary metabolites in tight clusters according to the *Citrus* species. In addition, the relationship between clusters was in agreement with the expected phylogenetic relationships among varieties, showing a perfect separation of the represented groups, *C. aurantium* L. and *C. reticulate* Blanco (Figure 4A). Nevertheless, by representing other combinations of components, the model is chaotic in differentiating harvesting period-related varieties within a group. In general, varieties were grouped according to genotype, but not harvesting period.

It seemed clear that the development stage had an influence on the fruit secondary metabolite composition as shown in previous research. Accordingly, the 37 *Citrus* samples were analyzed by chemotaxonomic classification using the 79 secondary metabolites as reference markers. HCA showed that those *Citrus* samples were divided into four branches (QP, CP, ZS and ZQ) according to their secondary metabolite chemotaxonomy (Figure 4B). Compared with primary metabolites, the HCA results of secondary metabolites showed different results for the chemotype-based classification methods, which reflected how the secondary metabolites play an important role in the chemotaxonomic classification of the *Citrus* harvesting period.

#### 2.4.2. Potential Chemotaxonomic Markers Associated with Primary Metabolites

A two-stage ROC curve analysis was carried out to identify potential markers associated with species. ROC analysis is a useful tool for evaluating the accuracy of a statistical model (e.g., logistic regression, linear discriminate analysis). The area under the ROC curve is a summary measure that essentially averages diagnostic accuracy [35].

As a result, 48 altered primary metabolites, with areas under the ROC curves ranging from 0.85–1 (Figure S1, Table 1 and Table S3), were considered to show the greatest diagnostic accuracy. Subsequently, an ROC curve-based model was established to assess the integrated predictive power of the combined 48 altered metabolites to distinguish *C. aurantium* L. from *C. reticulate* Blanco. The AUC value of the established model was 1.000, which showed a good ability for discriminating the two different *Citrus* species. Thus, those 48 altered metabolites (Table 1) can be defined as potential primary chemotaxonomic markers that are associated with different species.

#### 2.4.3. Potential Chemotaxonomic Markers Associated with Secondary Metabolites

**Figure 5:**
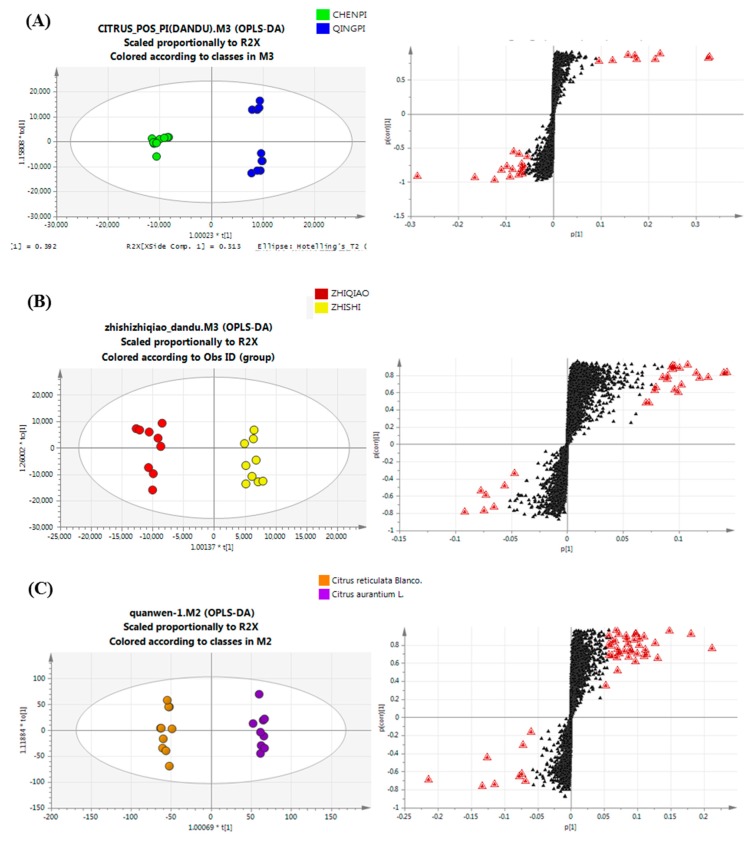
OPLS-DA score plots and loading S-plots of metabolites analyzed by UPLC-Q-TOF/MS in *Citrus* samples with the QP/CP group, ZS/ZQ group and CR/CA group. (**A**) Qingpi vs. Chenpi group; (**B**) Zhishi vs Zhiqiao group; (**C**) *Citrus reticulate* Blanco vs. *Citrus aurantium* L. group. The most differential compounds are marked with red triangles.

In this study, a detailed analysis of potential markers was conducted to determine the chemotype-features of these closely-related TCMs and the associations between the species and ripening stages of *Citrus* species [36]. To identify the metabolites that are significantly affected by ripening stages, OPLS-DA modeling was performed on the profiling datasets. The model separated young samples from ripe samples of *Citrus* by discriminating, according to their difference in light intensity. The OPLS-DA model of group QP/CP and ZS/ZQ explained (R^2^ = 0.812 and 0.886, respectively) and predicted (Q^2^ = 0.991 and 0.935, respectively) the total variance [37]. S-plots (Figure 5A,B) were constructed by presenting covariance (*p*) against correlation (pcorr), and the potential chemotaxonomic markers for the separation of shading effects were obtained by filtering with the variables that had an influence on the projection (VIP) > 1 and *p* < 0.05 in the statistical analysis. VIP was a weighted sum of squares of the PLS weight, and a value >1 was generally used as a criterion to identify the variables that were important to the model. Although many of the significantly differing components remained unknown, a total of 50 potential biomarkers was identified from the PLS-DA and S-plot as chemotaxonomic markers for ripening stages. The *p*-values of potential markers between groups (expressed as QP/CP and ZS/ZQ in Table 2) were calculated from their peak intensity to show the effects of different developmental stages of *Citrus*. As a result, flavones played a significantly important role in discriminating the ripening stage of *Citrus*; for example, sinensetin decreased and rutin increased in ripe fruits.

To explore the ripening effect by development stages, another multivariate statistical analysis was performed on the datasets from the two different species. Again, the two groups of samples were well separated by discriminating according to their differences. The OPLS-DA model explained more than 80% (R^2^) and predicted more than 95% (Q^2^) of the total variances. Analysis of the S-plot (Figure 5C) showed that most coumarins increased in the *C. aurantium* L. species, while flavonol, flavonol glycosides and anthocyanin increased in *C. reticulate* Blanco. A total of 57 potential biomarkers were identified as species chemotaxonomic markers, with a *p*-value between the group (*C. aurantium* L. and *C. reticulate* Blanco.), which is shown in Table 2.

Next, the PLS-DA-based receiver operating characteristic (ROC) curve was performed on each group to further find specific chemotaxonomic markers. This contribution map allowed comparisons between contrasting species groups and ripening stage groups (Figure S2), and it highlighted the potential regulatory nodes involved in different group variability by ROC curve analysis. In general, metabolites with areas under the ROC curves ranging from 0.85–1 are marked in the figure. As is shown, 25, 34 and 47 metabolites have been marked for QP/CP, ZS/ZQ and species group again, which is shown in Table 2. The AUC value of the established model is 0.934, 1.000 and 0.984 (Figure S2), which showed a good ability for discriminating.

### 2.5. The Pathway Analysis Associated with Chemotaxonomic Markers

Pathway analysis has been used to determine vegetative metabolic regulation that chiefly affected the following pathways: flavone and flavonol biosynthesis; flavonoid biosynthesis; anthocyanin biosynthesis; glyoxylate and dicarboxylate metabolism; alanine, aspartate and glutamate metabolism; starch and sucrose metabolism; amino sugar and nucleotide sugar metabolism; citrate cycle (TCA cycle); pyrimidine metabolism; pentose and glucuronate interconversions; lysine biosynthesis; cysteine and methionine metabolism; butanoate metabolism; beta-alanine metabolism; phenylalanine, tyrosine and tryptophan biosynthesis; nicotinate and nicotinamide metabolism; glycerophospholipid metabolism; cyanoamino acid metabolism; pyrimidine metabolism; carbon fixation pathways in photosynthetic organisms metabolism; and phenylpropanoid biosynthesis.

**Figure 6:**
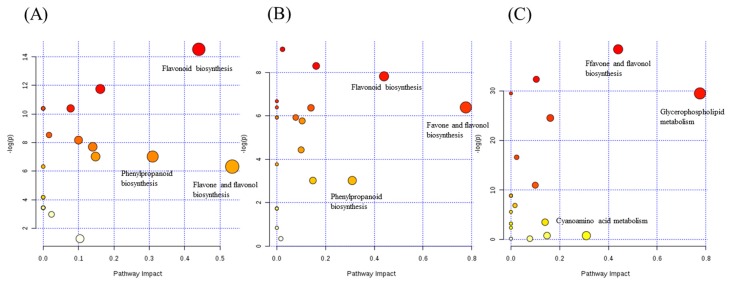
Pathway analysis of the identified metabolites. Each point represents one metabolic pathway; the size of the dot and shades of color represent a positive correlation with the impaction of the metabolic pathway. (**A**) The pathway analysis of the identified metabolites of the QP/CP group; (**B**) the pathway analysis of the identified metabolites of the ZS/ZQ group; (**C**) the pathway analysis of the identified metabolites of the CR/CA group.

Based on the primary metabolites and secondary chemotaxonomic markers, a comprehensive metabolic network for the discrimination of the species and ripening stages of *Citrus* fruits was mapped by KEGG (Kyoto Encyclopedia of Genes and Genomes) and MetaboAnalyst 3.0 [38] (shown in Figure 6A–C). Twenty-four metabolic pathways were disturbed in the QP/CP group, ZS/ZQ group and CA/CS group. The top three metabolic pathways with an impact value >0.2 influencing the species were glycerophospholipid metabolism, cyanoamino acid metabolism and flavone and flavonol biosynthesis; at the same time, three of the metabolic pathways were considered to be the most pertinent to the ripening stages in both the QP/CP and ZS/ZQ groups, including flavone and flavonol biosynthesis, flavonoid biosynthesis and phenylpropanoid biosynthesis.

#### 2.5.1. The PB Test of the Analysis Factor

A PB design is utilized to find experimental designs for investigating the dependence of some measured quantity on a number of independent variables to minimize the variance of the estimates of these dependencies using a limited number of experiments [39]. It was performed to investigate which factors significantly affected the different markers, as well. The two factors that we discussed below were assessed, including species (A) and ripening stages (B) (shown in Table S4). The contents of analyses were determined to be the influencing parameters of the markers. Main effects plots were used to examine differences between mean levels for those factors. Different levels of a factor affected the response differently. In main effect plots, if the line were parallel to the *x*-axis, there was no effect in reality. Conversely, this means a main effect. The steeper the slope of the line, the greater the magnitude of the main effect was observed. As shown in Figure S3, two factors seem to affect the content of the analyses because the lines are not horizontal. The slopes of the species and ripening stages curves in these graphs are all proportional to the absolute value of the estimated effects and the factors that have a significant effect on the response. Different chemotaxonomic markers have different effects on species and ripening stage.

#### 2.5.2. Analyzing a Wide Range of Secondary Metabolites to Understand the Metabolic Network

**Figure 7:**
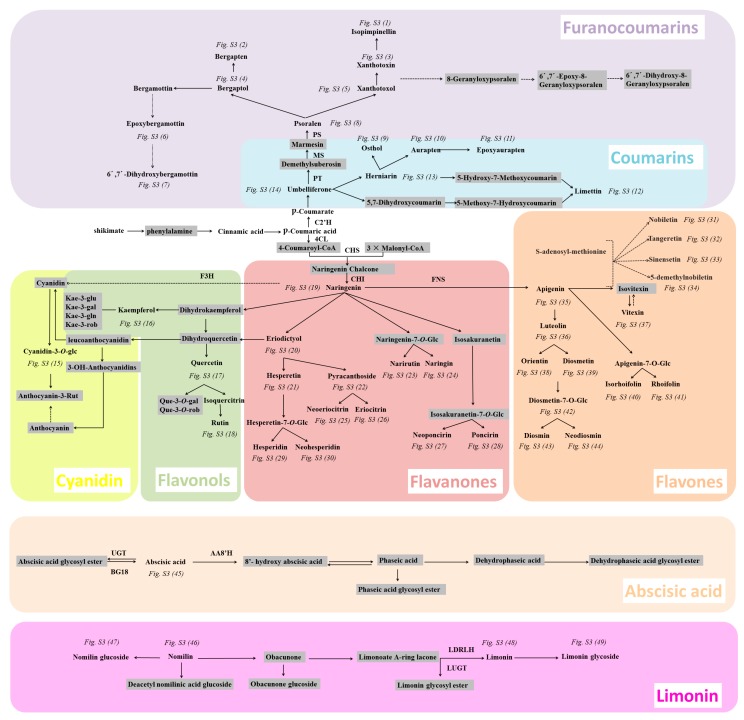
Comparison of the biosynthesis and accumulation of metabolites between *Citrus* samples. A simplified polyphenol biosynthetic pathway, starting from phenylalanine, was constructed on the basis of ‘omics’ data from previous studies on *Citrus*. The PB test was performed to investigate which factors significantly affected the different markers and is shown in Figure S3. Abbreviations for the main enzymes: CHS, chalcone synthase; CHI, chalcone isomerase; C2′H, *cis*-coumaroyl CoA 20-hydroxylase; C4H, cinnamate-4-hydroxylase; 4CL, 4-coumarate CoA ligase; F3H, flavanone 3-hydroxylase; FNS, flavone synthase; MS, marmesin synthase; PAL, phenylalanine ammonia lyase; PT, prenyltransferase; PS, psoralen synthase; BG18, beta-glucosidase 18; UGP, UDP-glucosyl transferase; AA8′H, abscisic acid 8′ hydroxylase; LDRLH, limonoid D-ring lactone hydrolase; LUGT, limonin UDP-glycosyl transferase. Compounds in grey are unidentified.

Flavonoids: This class of metabolites has great bioactivities, such as antioxidant activity, antifungal activity, antiparasitic activity and the beneficial health properties of *Citrus*. Indeed, the high radical scavenging activity in *Citrus* has been almost rarely associated with flavonoids and other phenolic constituents. In this study, from the same flavanone core, several following derivatives were identified by substitution with methyl groups or hexose moieties: naringin, hesperidin, narirutin, neohesperidin and eriodictyol (Figure 7 and Table 2). Among this group, the most widespread compounds were hesperidin, narirutin, sakuranetin and eriodictyol 7-*O*-neohesperidoside. These flavanone metabolites appeared to be found at higher concentrations in *C. aurantium* L. species. Regarding the ripening stage, hesperetin, hesperidin, neohesperidin and neoeriocitrin were marked as markers. The synthesis of flavonoid has been reported to start from the flavanone naringenin by continuous transfer of glycosyl groups (a first step by which glucose is transferred to oxygen in position 7, generating a 7-*O*-glucoside). In turn, a 1,6 rhamnosyl transferase renders the hesperidosides (or rutinosides) hesperidin and narirutin. Conversely, the action of 1,2 rhamnosyl transferase on flavanone 7-*O*-hexosides generates the neohesperidosides neohesperidin and naringin [40].

Flavone and flavonols: Flavones and flavonols synthesized from the same flavanone naringenin by hydroxylation include isorhamnetin, kaempferol and quercetin. One of the most important flavonols, rutin, has shown the highest accumulation in immature samples, which was the most different from other flavonols. This may be representative of a better flavonoid 3-monooxygenase activity in these imam ture samples. Moreover, isorhamnetin 3-*O*-rutinoside also showed a higher accumulation in immature samples and was derived from the addition of a hexose moiety to oxygen at position 3 catalyzed [40]. In our investigation, the variation trends of flavonol/flavone glycosides were found to depend on both aglycone species and ripening stages (Figure 7). The flavonoid content, as observed for apigenin, luteolin, chrysoeriol, vitexin-2-Orhamnoside, diosmetin, tetramethyl-isoscutellarein, sinensetin, tangeretin, nobiletin, diosmin, neodiosmin, luteolin-3′-7-di-*O*-glucoside, diosmetin-7-*O*-Glucoside, 5-demethylnobiletin and vitexin, was significantly elevated in QP samples and especially in ZS samples. By contrast, orientin and myricetin had higher contents in mature samples, both in CP and ZQ samples. Almost all of the flavonoids were markers in both the ripened stage and in spices; however, polymethoxylated flavones, such as sinensetin, tangeretin and 5-demethylnobiletin, did not perform well in the identified species.

Regarding flavonoids, temperature has an important effect on the biosynthesis and accumulation of phenolics in many plant species. Sub-groups of flavonoids were shown to be differently affected, which might be plant species dependent. For instance, flavonol in tomato increased when the temperature was low (18–12 °C), whereas grape plants grew under high temperatures (30–35 °C) [41]. Zhang et al. reported that lower temperature decreased flavonols and their glycosides in tea species [42].

Coumarins: The pathway of coumarin biosynthesis was largely discovered during the 1960s and 1970s, with the help of tracer feeding experiments. Some research indicated that umbelliferon is derived from *cis*-coumaric acid, whereas coumarin originates from *cis*-cinnamic acid [43]. As the beginning of coumarin biosynthesis, umbelliferon plays an important role in both the discrimination of species and ripening stage. For other coumarins, aurapten, epoxyaurapten and limettin have been identified as reflecting the ripening stage and species, whereas herniarin, 5,7-dihydroxycoumarin and 5-methoxy-7-hydroxycoumarin were only ripening stage factors [44].

While coumarin biosynthesis has remained a black box, several enzymes of the furanocoumarin pathway were isolated and characterized. Umbelliferone, rather than coumarin, is also the parent compound of furanocoumarins, as was reported a long time ago [44]. It is first prenylated at the 8-position for angular furanocoumarins to yield psoralen as the furanocoumarin beginning. Furanocoumarin performed well in identifying *Citrus*. Xanthotoxol, bergaptol, psoralen, xanthotoxin and bergapten 6′-7′-dihydroxybergamottin were both markers of species and ripening stages, and almost all furanocoumarin had a higher content in the *C. aurantium* L. species.

Abscisic acid (ABA) and derivatives: The pathway starting from ABA has two major branches: the catabolic and conjugating branches. The catabolic branch starts with the transfer of ABA into 8′-hydroxy ABA, which is catalyzed by ABA 8′-hydroxylase and then isomerizes to phaseic acid (PA). This metabolite will further catabolize to dehydrophaseic acid (DPA). The conjugating branch involves the temporary storage of ABA into a glycosylated form, catalyzed by an UDP-ABA glycosyl transferase (Figure 7). Actually, in this research, ABA had a higher content in mature samples [45].

Limonoids and glycosides: Limonoids are highly oxygenated triterpenes that are present in Rutaceae and Meliaceae. These metabolites are derived from squalene, although the first true limonoid precursor is nomilin, which can be directly glucosylated by a limonoid UDP-glucosyl transferase or also deacetylated (Figure 7), rendering obacunone [45]. All of the limonoids had higher contents in the *C. aurantium* L. species, especially in ZQ samples. Limonin could be used as a marker of the ripening stages in our study, and three other limonoids were both markers of ripening stage and species.

The phenylpropanoid biosynthesis, flavone and flavonol biosynthesis and flavonoid biosynthesis pathways are the most critical pathways for the synthesis of characteristic phenolic metabolites, including flavones, flavonols, flavanones and their glycosides, abscisic acid and procyanidins. Flavonoids have been reported to be lower in *Citrus* that is cultured in low temperature and low sun exposure conditions. Herein, we mapped the changes of metabolites involved in these pathways in Figure 7. The level of shikimate, which is a substrate of the phenylpropanoid biosynthesis pathway, was lower in CP and ZQ than in QP and ZS. At the same time, downstream metabolite products, such as *cis*-coumaric acid, abscisic acid, flavone glycosides, flavan-3-ols, procyanidins, coumarin and furanocoumarin, were significantly higher, which indicated more vigorous biosynthesis of phenolic secondary metabolites in QP and ZS than in CP and ZS. These results agreed with the study showing that the expression of structural genes encoding biosynthesis of flavonoids and the activity of some important enzymes increased under high light intensity and therefore led to a subsequent increase in the contents of flavonoids. This work paves the way for further analyses aiming to describe the control of the metabolism of phenolic metabolites in *Citrus* in response to genetic and ripening factors.

## 3. Materials and Methods

### 3.1. Chemicals and Reagents

HPLC-grade methanol was obtained from Fisher Scientific (Fair Lawn, NJ, USA). The deionized water was redistilled. Formic acid (HPLC grade, Lot. 095224) was obtained from MREDA Technology Inc. (Palo Alto, CA, USA). 7-hydroxycoumarin, bergapten and narirutin were obtained from Chengdu Must Bio-Technology Co., Ltd. (Chengdu, China). Hesperidin, naringin, apigetrin, kaempferitrin, diosmetin-7-*O*-glucoside and sinensetin were obtained from Shanghai Source Leaf Biological Technology Co., Ltd. (Shanghai, China). Eriocitrin, neohesperidin, naringenin, hesperetin, luteolin, rutin, quercetin, and tangeretin were obtained from Tianjin Mark Biological Technology Co., Ltd. (Tianjin, China). 5-femethylnobiletin, nobiletin, auraptene and bergamottin were obtained from Nanjing JingZhu Biological Technology Co., Ltd. (Nanjing, China). Rhoifolin, apigenin, scoparone, isorhamnetin-3-*O*-glucoside and diosmetin were purchased from the National Institute for Control of Biological and Pharmaceutical Products of China. Poncirin, eriodictyol, xanthotoxol, acacetin, isosakuranetin, imperatorin, limonin, nomilin and abscisic acid were purchased from Beijing Fufan Biological Technology Co., Ltd. (Beijing, China). The purity of the standards was relatively high (i.e., higher than 98%).

### 3.2. Sample Preparation

#### 3.2.1. Reference Standard Solution

Reference standards were dissolved in 50% methanol (1.0 mg/mL) and then stored at 4 °C until analysis.

#### 3.2.2. Extraction of Plant Material

A total of 37 batches of four different *Citrus* medicinal plants were collected from the Jiangxi, Sichuan, Hunan, Hubei and Zhejiang provinces and Chongqing municipality in China. (Table 3) Dried voucher specimens were deposited at the Institute of Basic Theory, China Academy of Chinese Medical Sciences, Beijing, China. The samples used for ACQUITY UPLC HSS T3 column extract detection were pulverized and sifted through a 60-mesh sieve. The method of sample preparation established and validated in our previous study was used. Accurately weighed powder (1.0 g) was placed in a conical flask. The material was sonicated three times with 10 mL of 50% MeOH for 30 min. The extraction solutions were filtered. The supernatant solution was combined and filtered through a 0.22-μm filter membrane. Each sample was extracted in parallel three times and then analyzed by UPLC-Q-TOF-MS.

As the samples for the ACQUITY UPLC BEH HILIC column method, the dried powder (1.0 g) was weighed accurately into a 10-mL conical flask with a stopper, and 10 mL water were added accurately. After weighing the filled flask accurately, ultrasonication (40 kHz) was carried out at room temperature for 30 min, then weighing again, and the same solvent (water) was added to compensate for the lost weight during the extraction as needed. After centrifugation (15,000× *g*, 10 min), the supernatant was stored at 4 °C and filtered through a 0.22-μm polytetrafluoroethylene filter before injection into the HILIC-UPLC-Q-TOF/MS system for analysis.

The extraction protocols and the repeatability data of samples have been reported in our pervious article [19,46].

### 3.3. Chromatographic Conditions

Chromatographic separation was performed on the Waters ACQUITY UPLC System (Waters Corp. Milford, MA, USA), equipped with a binary solvent delivery system and an autosampler. The extracts were performed on an ACQUITY UPLC HSS T3 column (100 mm × 2.1 mm, 1.8 μm). Water with 0.1% (*v*/*v*) formic acid and acetonitrile were used as Mobile Phases A and B, respectively, for chromatographic elution: from 0–2 min, Phase B was linearly increased from 0–20%, then linearly increased to 50% at 7.0 min and after that, linearly increased to 100% at 11 min and maintained for 3.0 min; Phase B was adjusted to 0% at 14.1 min for re-equilibration and maintained for 0.9 min. The total elapsed time required for a given chromatographic analysis was thus 15.0 min. The columns were maintained at 45 °C and eluted at a flow rate of 0.30 mL/min. The injection volume was 2 μL. The chromatograms of ACQUITY UPLC HSS T3 column have been put in the Supplemental Materials (Figure S8).

The extracts of samples were performed on the ACQUITY UPLC BEH HILIC column (100 mm × 2.1 mm, 1.7 μm). With the same Mobile Phases A and B with the ACQUITY UPLC HSS T3 column method, the gradient program for these samples was: from 0–1 min, 99% Phase B, then decreased at 70% B from 1–10 min, and remained at 99% Phase B from 10–15 min. The chromatograms of the ACQUITY UPLC BEH HILIC column have been put in the Supplemental Materials (Figure S9).

On the other side, we have demonstrated the chromatographic conditions in our pervious research [19].

### 3.4. Mass Spectrometry Condition

Mass spectrometry data were collected by using a Q-TOF analyzer in a Xevo G2 QTOF Mass system (Waters Corporation, Milford, MA, USA) in both positive and negative ion modes. The source temperature was set at 100 °C with a cone gas flow of 25.0 L/h, a desolvation gas flow of 800.0 L/h and a desolvation gas temperature of 400 °C. For the positive and negative ion modes, the capillary voltage was set to 3.0 kV, and the cone voltage was set to 40 V. The mass spectrometric data were collected in centroid mode from *m*/*z* 50–1200 with a scan time of 0.1 s and an interscan delay of 0.014 s over a 15.0-min analysis time. Leucine-enkephalin was used as the lock mass (*m*/*z* 556.2771 in positive mode and *m*/*z* 554.2615 in negative mode) at a concentration of 0.5 μg/mL with a flow rate of 30 μL/min The lock spray frequency was set at 20 s, and the lock mass data were averaged over 10 scans for correction. Compared with the negative ion mode, positive ion mode performed well in this study.

### 3.5. Data Processing and Statistical Analysis

Raw data files acquired from the UPLC-Q-TOF/MS analysis were imported into the Progenesis QI (Waters, Manchester, U.K.), which is used for UPLC-Q-TOF/MS data processing and database researching to pathway interrogation, to generate a peak table that included information on retention time, mass-to-charge ratio (*m*/*z*) and MS intensity of the metabolites in the sample. Additionally, the retention time tolerance and mass tolerance for the peak alignment were set to 0.20 and 0.05, respectively.

In order to perform the multivariate statistical analysis, the data we obtained from the UPLC-Q-TOF/MS including sample names (observations), the arbitrary compound index (*m*/*z*) and peak areas (variables) were introduced into the SIMCA-P 13.5 software (Umetric, Umeå, Sweden). The raw data were subjected to principal component analysis (PCA) to visualize general clustering, trends or outliers among the observations. Partial least-squares discriminant analysis (PLS-DA) was utilized to validate the PCA model and identify the differential metabolites. R^2^ represented the explanation capacity of the model (R^2^X and R^2^Y represent the fraction of the variance of X matrix and Y matrix), whereas Q^2^ suggested the predictive accuracy of the model. The cumulative values of R^2^X, R^2^Y and Q^2^ close to 1 meant an excellent model. The differential metabolites were selected by the statistically-significant threshold of the variable influence on projection (VIP) values. Heat-map analysis, pathway analysis and ROC curves were performed using MetaboAnalyst 3.0, which is an open bioinformatics website for metabolite data interpretation. The significance level of the metabolite differences between groups was calculated by Student’s *t*-test using the SPSS software (Version 22.0, International Business Machines Corp., Armonk, NY, USA). Results were considered significant when the *p*-value was less than 0.05.

Putative metabolites were first derived by searching the exact molecular mass data from redundant *m*/*z* peaks against the online HMDB (http://www.hmdb.ca/), PubMed (http://www. ncbi.nlm.nih.gov/pubmed/), LMSD (http://www.lipidmaps.org/), KEGG (http://www.kegg.jp/) and ChemSpider (http://www.chemspider.com) databases. A specific metabolite would be sieved out when a match with a difference between observed and theoretical mass was less than 5 ppm. Then, the metabolite molecular formula of matched metabolites was further identified by the isotopic distribution measurement.

## 4. Conclusions

In this study, a metabolomics-guided chemotaxonomic classification strategy was applied to investigate four closely-related *Citrus* TCMs. A UPLC-Q-TOF-MS method was employed for the analysis of primary and secondary metabolites from *Citrus* TCMs with different species and ripening stages. It indicated that different *Citrus* TCMs have different metabolic profiles depending on genotype-regulated primary/secondary metabolites and that the development stage influenced secondary metabolites, which could have an impact on *Citrus* nutritional properties for quality control and efficient clinical usage. Thus, the genotype is expected to be the major contributing factor in determining fruit compositional properties related to species; however, it is worth noting that different growth stages may give rise to new varieties that can also affect the fruit chemical composition. Moreover, multivariate statistical analysis coupled with MetaboAnalyst permits a comprehensive depiction of the variable chemotaxonomic markers and the elucidation of the biosynthesis mechanism of *Citrus* TCMs from different species and different ripening stages. The top three metabolic pathways with an impact value >0.2 influencing the species were glycerophospholipid metabolism, cyanoamino acid metabolism and flavone and flavonol biosynthesis. Meanwhile, three of the metabolic pathways were considered to be the most pertinent to the ripening stages in both the QP/CP and ZS/ZQ groups, including flavone and flavonol biosynthesis, flavonoid biosynthesis and phenylpropanoid biosynthesis.

## Figures and Tables

**Table 1:** Differential primary metabolites between the *Citrus reticulate* Blanco and *Citrus aurantium* L. group profile in positive and negative ion modes. GPA (glycerophosphatidic acid); MG (monogalactosyl); PE (phosphatidylethanolamine); DG (diacylglycerol); GPEtn (glycerophosphoethanolamine); PC (phosphatidylcholine); GPIns (Glycerophosphatidylinositol), GPSer (Glycerophosphatidylserine acid), GPGro (Glycerophosphatidylglycerol); UMP (Uridine monophosphate).

No.	Retention Time (RT)	Formula	[M + H]^+^ *m*/*z*		[M − H]^−^ *m*/*z*		Identification	*p* Value	AUC	Metabolic Pathways
			Detected	Mass Error	Detected	Mass Error				
M1	4.38	C_10_H_13_NO_2_	180.1024	1.9	-	-	3,5-Dimethylphenyl methylcarbamate	8.77 × 10^−9^	1.0000	Not known
M2	6.52	C_10_H_19_O_8_P	299.0892	0.7	-	-	GPA(5:0/2:0)/GPA(2:0/5:0)	0.0010	1.0000	Glycerophospholipid metabolism
M3	2.91	C_11_H_12_N_2_	173.1079	−0.8	171.1077	−1.6	1,2,3,4-Tetrahydro-beta-carboline	2.63 × 10^−6^	1.0000	Not known
M4	7.06	C_14_H_27_O_8_P	355.1523	−0.4	-	-	GPA(9:0/2:0)/GPA(2:0/9:0)	1.93 × 10^−9^	1.0000	Glycerophospholipid metabolism
M5	8.73	C_15_H_28_O_8_P	369.1681	−0.9	-	-	GPA(10:0/2:0)/GPA(2:0/10:0)	3.99 × 10^−7^	1.0000	Glycerophospholipid metabolism
M6	4.75	C_18_H_35_O_8_P	411.2168	4.9	-	-	GPA(10:0/5:0)/GPA(5:0/10:0)	1.37 × 10^−8^	1.0000	Glycerophospholipid metabolism
M7	9.71	C_18_H_36_O_4_	339.2514	2.6	-	-	MG(15:0/0:0/0:0)	3.95 × 10^−5^	1.0000	Not known
M8	9.32	C_19_H_35_O_8_P	423.2139	−0.7	-	-	GPA(14:1/2:0)/GPA(2:0/14:1)	1.00 × 10^−20^	1.0000	Glycerophospholipid metabolism
M9	4.32	C_19_H_36_O_4_	369.2695	2.5	-	-	MG(16:1/0:0/0:0)	3.45 × 10^−4^	1.0000	Not known
M10	6.53	C_19_H_36_NO_7_P	423.2410	−2.1	-	-	PE(14:1/0:0)	7.70 × 10^−9^	1.0000	Phosphatidylethanolamine biosynthesis
M11	9.95	C_19_H_38_O_4_	331.2850	2.0	-	-	MG(16:0/0:0/0:0)	1.94 × 10^−9^	1.0000	Not known
M12	2.73	C_20_H_37_O_8_P	437.2301	0.5	-	-	GPA(14:1/3:0)/GPA(3:0/14:1)	1.94 × 10^−4^	1.0000	Glycerophospholipid metabolism
M13	10.31	C_20_H_38_O_5_	359.2620	2.3	-	-	DG(8:0/9:0/0:0)/DG(9:0/8:0/0:0)	5.11 × 10^−5^	1.0000	Not known
M14	11.63	C_21_H_34_O_4_	351.2538	2.9	-	-	MG(18:4/0:0/0:0)	0.0020	1.0000	Not known
M15	10.03	C_21_H_44_NO_7_P	454.2783	0.3	452.2781	−0.3	PE(16:0/0:0)	1.69 × 10^−5^	1.0000	Phosphatidylethanolamine biosynthesis
M16	11.41	C_25_H_44_O_15_	585.2551	−3.6	-	-	DGDG(2:0/8:0)/DGDG(8:0/2:0)	0.0053	1.0000	DGDG synthase
M17	12.05	C_26_H_46_O_15_	599.2709	−3.3	-	-	DGDG(2:0/9:0)/DGDG(9:0/2:0)	4.64 × 10^−6^	1.0000	DGDG synthase
M18	10.99	C_26_H_49_O_8_P	521.3257	0.72	-	-	GPA(14:1/9:0)/GPA(9:0/14:1)	3.80 × 10^−7^	1.0000	Glycerophospholipid metabolism
M19	11.78	C_8_H_16_O_4_	177.1126	2.5	-	-	MG(5:0/0:0/0:0)	4.90 × 10^−10^	1.0000	Not known
M20	2.61	C_8_H_18_OS_2_	195.0694	1.4	193.0690	0.4	3-[(2-Mercapto-1-methylpropyl)thio]-2-butanol	2.78 × 10^−9^	1.0000	Not known
M21	5.36	C_8_H_6_O_2_	135.0446	3.9	133.0438	−1.8	Phthalide	0.022	1.0000	Not known
M22	0.87	C_8_H_9_NO_2_	152.0708	1.0	150.0707	0.4	2-Phenylglycine	3.94 × 10^−13^	1.0000	Not known
M23	8.54	C_26_H_52_O_8_P	-	-	524.6784	0.23	GPA(11:0/12:0)/GPA(12:0/11:0)	5.15 × 10^−4^	1.0000	Glycerophospholipid metabolism
M24	8.57	C_27_H_51_O_13_P	-	-	613.3005	1.7	GPIns(10:0/8:0)/GPIns(8:0/10:0)	1.01 × 10^−6^	1.0000	Not known
M25	8.57	C_19_H_37_O_8_P	-	-	424.4562	−3.3	GPA(10:0/6:0)/GPA(6:0/10:0)	7.03 × 10^−12^	1.0000	Glycerophospholipid metabolism
M26	8.44	C_13_H_25_O_8_P	-	-	339.1223	2.6	GPA(2:0/8:0)/GPA(8:0/2:0)	2.66 × 10^−8^	1.0000	Glycerophospholipid metabolism
M27	6.48	C_14_H_25_O_13_P	-	-	431.0977	4.0	GPIns(2:0/3:0)/GPIns(3:0/2:0)	2.14 × 10^−5^	1.0000	Glycerophospholipid metabolism
M28	10.78	C_25_H_46_O_10_	-	-	503.2850	−2.4	MGDG(14:1/2:0)/MGDG(2:0/14:1)	0.0010	1.0000	monogalactosyldiacylglycerol synthase
M29	0.92	C_5_H_10_N_2_O_3_	147.0618	0.0	145.0616	−1.7	Glutamine	0.0039	1.0000	Inosine monophosphate biosynthesis
M30	9.16	C_21_H_36_O_4_	353.2692	2.1	-	-	MG(18:3/0:0/0:0)	1.13 × 10^−13^	0.9941	Not known
M31	10.96	C_21_H_40_O_4_	357.3008	2.4	-	-	MG(18:1/0:0/0:0)	0.012	0.9941	Not known
M32	5.44	C_26_H_30_O_14_	567.1717	1.0	565.1703	−1.4	Mulberroside F	0.0040	0.9941	Not known
M33	4.12	C_10_H_10_O_2_	163.0758	2.2	161.0749	−3.4	4,5-Dihydro-1-benzoxepin-3(2H)-one	5.98 × 10^−8^	0.9912	Not known
M34	0.80	C_9_H_13_N_2_O_9_P	321.0289	0.7	323.0291	1.3	UMP	9.79 × 10^−15^	0.9912	Uridine monophosphate biosynthesis
M35	3.38	C_10_H_20_NO_8_P	314.1928	0.3	312.1916	−2.4	GPEtn(2:0/3:0)/GPEtn(3:0/2:0)	1.63 × 10^−8^	0.9883	Glycerophospholipid metabolism
M36	2.73	C_16_H_25_N_5_O_6_	384.1865	−3.3	-	-	Dihydrozeatin-*O*-glucoside	5.77 × 10^−5^	0.9854	Not known
M37	3.44	C_10_H_19_O_10_P	-	-	330.1346	1.9	GPGro(2:0/2:0)	8.11 × 10^−8^	0.9854	Glycerophospholipid metabolism
M38	3.26	C_11_H_22_NO_8_P	-	-	326.2290	−2.2	GPEtn(2:0/4:0)/GPEtn(4:0/2:0)	2.28 × 10^−9^	0.9795	Glycerophospholipid metabolism
M39	2.92	C_8_H_18_NO_7_P	-	-	270.1774	−1.2	PE(3:0/0:0)	4.11 × 10^−4^	0.9795	Phosphatidylethanolamine biosynthesis
M40	6.28	C_20_H_20_O_9_	405.1185	1.3	405.1174	−1.3	*cis*-resveratrol 4′-*O*-glucuronide	1.26 × 10^−7^	0.9737	Not known
M41	0.87	C_7_H_14_N_2_O_3_	175.0920	−4.5	173.0929	−1.4	Theanine	1.21 × 10^−8^	0.9737	Theanine synthetase
M42	0.67	C_4_H_8_N_2_O_3_	133.0461	−1.3	131.0458	−3.0	Asparagine	1.58 × 10^−13^	0.9503	Asparagine synthase
M43	9.15	C_18_H_34_O_4_	315.2357	1.5	-	-	MG(15:1/0:0/0:0)	0.016	0.9386	Not known
M44	9.67	C_26_H_50_NO_7_P	520.3330	1.4	-	-	PC(18:2/0:0)	5.44 × 10^−5^	0.9211	Phosphatidylcholine biosynthesis
M45	3.37	C_20_H_23_NO_9_	422.1449	0.2	-	-	5-*O*-*cis*-Coumaroylnigrumin	7.01 × 10^−4^	0.9152	Not known
M46	10.50	C_26_H_52_NO_7_P	522.3487	1.1	-	-	PC(18:1/0:0)/PE(P-16:0/5:0)/PE(P-18:0/3:0)	2.20 × 10^−5^	0.9006	Phosphatidylcholine biosynthesis
M47	2.86	C_7_H_10_O_5_	175.0454	−0.4	173.0456	0.4	Shikimic acid	1.73 × 10^−5^	0.8918	TCA cycle
M48	0.81	C_4_H_7_NO_4_	133.0300	−1.4	133.0303	0.7	Aspartic acid	1.44 × 10^−4^	0.8801	TCA cycle

**Table 2:** Differential secondary metabolites between Chenpi (CP)/Qingpi (QP), Zhiqiao (ZQ)/Zhishi (ZS) and *Citrus aurantium* L. (CA)/ *Citrus reticulate* Blanco. (CR) group profiles in positive and negative ion modes.

No.	RT (min)	Formula	[M + H]^+^ *m*/*z*		[M − H]^−^ *m*/*z*		Mw (Da)	Identification	VIP (*p*-Value)	AUC
			Detected	Mass Erorr	Detected	Mass Erorr				
Flavones and flavones glycoside										
1	3.37	C_15_H_10_O_8_	319.0446	−0.6	317.0447	−0.3	318.0448	Myricetin	1.22 (2.08 × 10^−5^) ^a^, 1.17 (2.99 × 10^−5^) ^c^	0.9778 ^a^
2	3.83	C_21_H_20_O_10_	433.0988	1.2	431.0978	−1.1	432.0983	Vitexin	1.78 (1.46 × 10^−4^) ^c^	0.9181 ^c^
3 *	3.86	C_22_H_22_O_12_	479.1185	0.2	477.1175	−1.9	478.1184	Isorhamnetin-3-*O*-glucoside	1.24 (6.37 × 10^−4^) ^a^, 2.11 (0.019) ^c^	0.9000 ^a^
4 *	3.93	C_22_H_22_O_11_	463.1156	−1.1	461.1183	4.7	462.1161	Diosmetin-7-*O*-glucoside	2.23(9.87 × 10^−9^) ^b^	1.0000 ^b^
5	4.09	C_27_H_30_O_14_	579.1630	−1.0	577.1652	2.8	578.1636	Isorhoifolin	1.48 (0.005) ^a^, 1.40 (0.031) ^c^	0.9111 ^a^
6	4.13	C_21_H_20_O_11_	449.1003	−0.4	447.0989	−3.6	448.1005	Orientin	2.68 (1.52 × 10^−9^) ^a^, 1.25 (0.017) ^c^	1.0000 ^a^
7	4.18	C_26_H_28_O_14_	565.1475	−0.7	563.1483	0.7	564.1479	Apiin	13.68 (9.24 × 10^−15^) ^c^	1.0000 ^c^
8 *	4.2	C_27_H_30_O_14_	579.1626	−1.7	577.1655	3.3	578.1636	Rhoifolin	9.35 (3.86 × 10^−11^) ^c^	1.0000 ^c^
9	4.28	C_28_H_32_O_15_	609.1745	0.7	607.1720	−3.5	608.1741	Diosmin	1.32 (0.013) ^a^, 2.52 (0.0021) ^b^, 4.39 (8.97 × 10^−10^) ^c^	0.9012 ^b^, 0.9737 ^c^
10	4.37	C_28_H_32_O_15_	609.1745	0.7	607.1720	−3.5	608.1741	Neodiosmin	4.52 (1.80 × 10^−5^) ^b^, 5.89 (3.21 × 10^−10^) ^c^	1.0000 ^b^, 0.9971 ^c^
11	4.42	C_27_H_30_O_16_	611.1526	−1.3	609.1530	−0.7	610.1534	Luteolin-3′,7-di-*O*-glucoside	7.28 (0.0033) ^b^, 10.19 (7.85 × 10^−12^) ^c^	0.8642 ^b^, 0.9883 ^c^
12	4.47	C_21_H_20_O_11_	449.1009	0.9	447.0989	−3.6	448.1005	Cynaroside	4.52 (0.0035) ^b^, 8.87 (1.83 × 10^−13^) ^c^	0.9912 ^c^
13	4.47	C_28_H_32_O_14_	593.1786	−1.0	591.1785	−1.2	592.1792	Linarin	1.20 (0.049) ^b^, 2.0 (1.19 × 10^−12^) ^c^	0.9883 ^c^
14	5.21	C_16_H_12_O_6_	301.0629	−1.7	299.0621	−4.3	300.0634	Chrysoeriol	7.02 (7.09 × 10^−4^) ^b^, 12.07 (9.40 × 10^−13^) ^c^	0.9136 ^b^, 1.0000 ^c^
15	5.21	C_30_H_18_O_10_	539.0896	−0.7	537.0911	2.0	538.0900	Amentoflavone	1.93 (6.36 × 10^−5^) ^b^, 2.21 (2.30 × 10^−7^) ^c^	0.9630 ^b^, 1.0000 ^c^
16 *	5.51	C_21_H_20_O_10_	433.1040	−3.9	431.1037	−4.6	432.1057	Apigetrin	1.52 (8.6 × 10^−4^) ^b^, 4.66 (1.83 × 10^−17^) ^c^	1.0000 ^c^
17	5.51	C_21_H_20_O_11_	449.1015	2.2	447.0989	−3.6	448.1005	Luteolin-4′-*O*-glucoside	1.68 (0.0019) ^b^, 3.96 (1.17 × 10-15) ^c^	0.8889 ^b^, 1.0000 ^c^
18	5.51	C_27_H_30_O_15_	595.1589	0.8	593.1577	−1.2	594.1584	Saponarin	2.44 (0.0012) ^b^, 5.54 (1.04 × 10^−15^) ^c^	0.9136 ^b^, 1.0000 ^c^
19 *	5.58	C_15_H_10_O_6_	287.0476	−0.3	285.0477	0.0	286.0477	Luteolin	1.39 (0.0016) ^a^, 2.46 (0.035) ^b^, 8.67 (3.48 × 10^−17^) ^c^	1.0000 ^c^
20	6.3	C_15_H_10_O_5_	271.0526	−0.7	269.0524	−1.5	270.0528	Apigenin	3.43 (0.032) ^a^, 2.27 (7.13 × 10^−4^) ^c^	0.9667 ^a^, 0.8889 ^c^
21 *	6.58	C_16_H_12_O_6_	301.0637	1.0	299.0630	−1.3	300.0634	Diosmetin	3.42 (6.16 × 10^−4^) ^b^, 2.37 (0.0020) ^c^	0.8778 ^a^, 0.9753 ^b^, 0.8597 ^c^
22 *	6.95	C_16_H_12_O_5_	285.0678	−2.5	283.0690	1.8	284.0685	Acacetin	1.24 (0.0030) ^b^, 1.49 (3.95 × 10^−7^) ^c^	0.8667 ^a^, 0.8642 ^b^, 1.0000 ^c^
23 *	7.75	C_20_H_20_O_7_	373.1212	0.8	371.1208	−0.3	372.1209	Sinensetin	8.98 (0.0083) ^a^, 4.79 (0.016) ^b^	-
24 *	8.35	C_21_H_22_O_8_	403.1320	1.2	-	-	402.1315	Nobiletin	16.79 (0.025) ^a^, 14.60 (0.0087) ^b^	-
25	8.38	C_19_H_18_O_6_	343.1108	1.5	-	-	342.1103	Tetramethyl-isoscutellarein	9.15 (0.0059) ^a^, 9.34 (0.035) ^b^, 4.48 (0.046) ^c^	-
26 *	8.88	C_20_H_20_O_7_	373.1201	−2.1	-	-	372.1209	Tangeretin	17.13 (0.015) ^a^, 16.34 (0.0094) ^b^	0.8642 ^b^
27 *	9.31	C_20_H_20_O_8_	389.1162	1.0	-	-	388.1158	5-demethylnobiletin	11.83 (0.0045) ^a^, 8.46 (1.63 × 10^−6^) ^b^, 3.89 (0.050) ^c^	0.8667 ^a^, 0.9260 ^b^
Flavanones and flavanone glycoside										
28 *	3.71	C_27_H_32_O_15_	597.1745	0.7	595.1745	0.7	596.1741	Eriocitrin	2.71 (2.70 × 10^−8^) ^c^	0.9357 ^c^
29	3.73	C_21_H_22_O_11_	451.1156	−1.3	449.1164	0.4	450.1162	Pyracanthoside	2.85 (9.22 × 10^−16^) ^c^	0.9971 ^c^
30	3.74	C_28_H_34_O_14_	595.1940	−1.5	593.1945	−0.7	594.1949	Neoponcirin	2.74 (0.0012) ^a^, 1.58 (0.0047) ^c^	0.8778 ^a^
31	3.82	C_27_H_32_O_15_	597.1746	0.8	595.1735	−1.0	596.1741	Neoeriocitrin	1.73 (0.050) ^b^, 3.88 (5.98 × 10^−11^) ^c^	1.0000 ^c^
32 *	3.84	C_28_H_34_O_14_	595.1935	−2.3	593.1955	1.0	594.1949	Poncirin	1.38 (3.97× 10^−11^) ^a^, 1.24 (5.44 × 10^−8^) ^c^	1.0000 ^a^, 0.9620 ^c^
33 *	4.1	C_27_H_32_O_14_	581.1786	−1.0	579.1780	−2.1	580.1792	Narirutin	2.06 (0.010) ^a^, 1.07 (0.044) ^b^, 5.38 (5.12 × 10^−20^) ^c^	-
34 *	4.26	C_27_H_32_O_14_	581.1802	1.7	579.1797	0.9	580.1792	Naringin	1.43 (0.013) ^a^, 2.52 (0.032) ^b^, 13.64 (5.97 × 10^−19^) ^c^	1.0000 ^c^
35 *	4.34	C_28_H_34_O_15_	611.1882	−2.5	609.1896	−0.2	610.1897	Hesperidin	1.39 (0.031) ^a^, 2.00 (0.0092) ^b^, 2.89 (2.81 × 10^−5^) ^c^	0.9912 ^c^
36 *	4.49	C_28_H_34_O_15_	611.1889	−1.3	609.1905	1.3	610.1897	Neohesperidin	1.91 (0.041) ^a^, 7.92 (0.0082) ^b^, 16.60 (5.1 × 10^−5^) ^c^	-
37	4.69	C_16_H_14_O_6_	303.0792	0.7	301.0785	−1.7	302.0790	Homoeriodictyol	4.85 (5.43 × 10^−4^) ^b^, 8.93 (3.55 × 10^−12^) ^c^	0.8778 ^a^, 0.9506 ^b^, 0.9912 ^c^
38 *	4.82	C_15_H_12_O_6_	289.0628	−2.1	287.0628	−2.1	288.0634	Eriodictyol	5.99 (8.49 × 10^−14^) ^c^	1.0000 ^c^
39	5.51	C_16_H_14_O_5_	287.0835	−2.1	285.0854	4.5	286.0841	Sakuranetin	1.46 (0.022) ^a^, 2.47 (0.035) ^b^, 9.26 (3.48 × 10^−17^) ^c^	1.0000 ^c^
40 *	6.3	C_15_H_12_O_5_	273.0673	−4.4	271.0691	2.2	272.0685	Naringenin	4.64 (0.028) ^a^, 8.95 (9.7 × 10^−4^) ^b^, 9.37 (2.30 × 10^−5^) ^c^	0.9240 ^c^
41 *	6.62	C_16_H_14_O_6_	303.0795	1.7	301.0785	−1.7	302.0790	Hesperetin	2.31 (0.0011) ^a^, 8.57 (0.049) ^b^, 7.07 (3.52 × 10^−13^) ^c^	-
Flavonols										
42 *	3.39	C_27_H_30_O_14_	579.1630	−1.0	577.1629	−1.2	578.1636	Kaempferitrin	1.66 (2.16 × 10^−7^) ^a^, 1.74 (0.025) ^b^	1.0000 ^a^, 0.8518 ^b^
43 *	3.67	C_27_H_30_O_16_	611.1543	1.5	609.1541	1.1	610.1534	Rutin	2.59 (6.42 × 10^−9^) ^a^, 2.08 (1.22 × 10^−4^) ^c^	1.0000 ^a^, 0.8742 ^c^
44 *	5.60	C_15_H_10_O_7_	303.0425	−0.7	301.0425	−0.7	302.0427	Quercetin	2.56 (2.77 × 10^−4^) ^c^	0.9506 ^b^, 0.8684 ^c^
Cyanidin										
45	2.98	C_21_H_21_ClO_11_	485.0782	2.1	483.0770	−0.4	484.0772	Cyanidin-3-*O*-glucoside	2.42 (0.0061) ^a^	1.0000 ^a^
Coumarins										
46	3.46	C_11_H_10_O_4_	207.0577	−1.0	205.0585	2.9	206.0579	Limettin	3.06 (1.10 × 10^−8^) ^a^, 2.08 (6.4 × 10^−4^) ^c^	1.0000 ^a^
47 *	4.35	C_9_H_6_O_3_	163.0312	−3.1	161.0310	−4.3	162.0317	Umbelliferone	1.96 (7.53 × 10^−5^) ^b^, 1.89 (2.86 × 10^−5^) ^c^	1.0000 ^b^, 0.9270 ^c^
48 *	5.34	C_11_H_10_O_4_	207.0570	−4.4	-	-	206.0579	Scoparone	1.09 (0.0077) ^b^	-
49	6.15	C_20_H_24_O_4_	329.1681	2.1	-	-	328.1674	5-Geranyloxy-7-Methoxycoumarin	2.17 (0.0089) ^a^, 2.04 (0.0212) ^b^	0.9012 ^b^
50	7.85	C_19_H_22_O_4_	315.1507	−3.5	-	-	314.1518	Epoxyaurapten	1.16 (0.0075) ^a^, 4.62 (1.73 × 10^−7^) ^b^, 4.12 (6.73 × 10^−8^) ^c^	0.8667 ^a^, 1.0000 ^b^, 0.9737 ^c^
51	9.60	C_15_H_16_O_3_	245.1102	1.2	-	-	244.1099	Osthol	3.6 (0.016) ^b^, 4.12 (2.12 × 10^−8^) ^c^	1.0000 ^c^
52	10.67	C_19_H_22_O_3_	299.1571	0.7	-	-	298.1569	Aurapten	4.76 (2.16 × 10^−5^) ^b^, 5.17 (4.83 × 10^−7^) ^c^	0.9753 ^b^
53 *	11.00	C_19_H_22_O_3_	299.1565	−1.3	297.1568	−0.3	298.1569	Auraptene	10.50 (1.39 × 10^−4^) ^b^, 12.57 (6.83 × 10^−8^) ^c^	0.8556 ^a^, 0.9506 ^b^, 1.0000 ^c^
Furanocoumarins										
54	4.56	C_17_H_18_O_7_	335.1053	0.3	-	-	334.1052	Byakangelicin	1.23 (3.19 × 10^−4^) ^b^, 1.33 (0.0016) ^c^	0.8889 ^a^, 1.0000 ^b^
55	5.31	C_16_H_16_O_6_	305.0941	−2.0	-	-	304.0947	Heraclenol	2.47 (2.79 × 10^−6^) ^b^, 1.01 (0.0015) ^c^	1.0000 ^b^, 0.9591 ^c^
56	5.51	C_16_H_14_O_5_	287.0845	1.4	-	-	286.0841	Heraclenin	1.05 (0.022) ^a^, 5.37 (1.41 × 10^−17^) ^c^	-
57	5.72	C_16_H_16_O_6_	305.0940	−2.3	-	-	304.0947	Oxypeucedanin hydrate	1.01 (0.0016) ^c^	0.9532 ^c^
58	6.75	C_11_H_6_O_3_	187.0318	0.5	-	-	186.0317	Psoralen	1.27 (0.0078) ^b^, 1.72 (5.99 × 10^−9^) ^c^	0.8642 ^b^
59	7.06	C_12_H_8_O_4_	217.0420	−0.9	215.0418	−1.9	216.0422	Xanthotoxin	1.01 (2.09× 10^−7^) ^b^,1.02 (3.07 × 10^−7^) ^c^	0.9971 ^c^
60	7.06	C_21_H_22_O_5_	355.1472	1.4	-	-	354.1467	Epoxybergamottin	1.00 (0.0305) ^a^, 6.77 (7.82 × 10^−7^) ^b^, 8.57 (2.94 × 10^−9^) ^c^	0.8667 ^a^, 1.0000 ^b^, 1.0000 ^c^
61 *	7.43	C_12_H_8_O_4_	217.0421	−0.5	-	-	216.0422	Bergapten	3.07 (0.019) ^b^, 2.82 (0.0063) ^c^	0.9673 ^c^
62	7.48	C_13_H_10_O_5_	247.0531	1.2	-	-	246.0528	Isopimpinellin	1.03 (2.10 × 10^−4^) ^b^	0.9591 ^c^
63 *	7.89	C_11_H_6_O_4_	203.0274	4.0	201.0270	2.0	202.0266	Xanthotoxol	6.44 (0.027) ^a^, 2.73 (2.82× 10^−7^) ^b^, 6.43 (1.6× 10^−4^) ^c^	-
64	7.89	C_17_H_16_O_5_	301.1007	3.0	-	-	300.0998	Cnidilin	1.01 (6.16 × 10^−4^) ^b^, 1.09 (0.0020) ^c^	0.8778 ^a^, 0.9753 ^b^, 0.8597 ^c^
65	8.22	C_21_H_24_O_6_	373.1581	2.1	-	-	372.1573	6′-7′-dihydroxybergamottin	1.11 (4.49 × 10^−5^) ^b^, 1.02 (5.61 × 10^−6^) ^c^	0.8778 ^a^, 0.9753 ^b^, 0.8655 ^c^
66	8.24	C_16_H_14_O_5_	287.0832	−3.1	-	-	286.0841	Oxypeucedanin	1.46 (0.016) ^a^, 2.04 (0.0083) ^b^, 1.94 (0.0047) ^c^	0.8778 ^a^
67	8.45	C_17_H_16_O_6_	317.0956	2.8	-	-	316.0947	Byakangelicol	1.05 (0.0063) ^b^	0.9383 ^b^
68 *	9.27	C_16_H_14_O_4_	271.0895	1.1	-	-	270.0892	Imperatorin	1.62 (0.0060) ^b^, 1.52 (7.13 × 10^−4^) ^c^	0.8889 ^c^
69	9.54	C_17_H_16_O_5_	301.0997	−0.3	-	-	300.0998	Phellopterin	1.14 (7.09 × 10^−4^) ^b^	0.9136 ^b^
70	9.76	C_16_H_14_O_4_	271.0892	0.0	-	-	270.0892	Isoimperatorin	1.84 (0.032) ^b^, 1.67 (0.023) ^c^	-
71	10.37	C_21_H_22_O_5_	355.1465	−0.6	353.1460	−2.0	354.1467	Cnidicin	1.39 (0.017) ^b^, 2.61 (3.33E^−7^) ^c^	1.0000 ^c^
72	10.87	C_21_H_22_O_4_	339.1513	−1.5	-	-	338.1518	8-geranyloxypsoralen	1.07 (0.0078) ^b^	0.8642 ^b^, 0.9357 ^c^
73	11.26	C_11_H_6_O_4_	203.0267	0.5	201.0271	2.5	202.0266	Bergaptol	3.70 (0.046) ^a^, 3.14 (1.16 × 10^−4^) ^b^, 3.70 (5.51 × 10^−7^) ^c^	0.9506 ^b^
74 *	11.56	C_21_H_22_O_4_	339.1522	1.2	337.1510	0.5	338.1518	Bergamottin	1.47 (6.11 × 10^−5^) ^b^, 1.42 (2.14 × 10^−5^) ^c^	0.9630 ^b^, 1.0000 ^c^
Limonin										
75	3.93	C_32_H_42_O_14_	651.2430	−1.2	649.2441	0.5	650.2438	Limonin glycoside	4.45 (3.45 × 10^−5^) ^a^, 3.01 (0.0055) ^b^	-
76 *	7.82	C_26_H_30_O_8_	471.2033	0.4	469.2035	0.9	470.2031	Limonin	3.01 (0.0023) ^a^, 5.66 (1,8 × 10^−5^) ^b^, 9.63 (3.04 × 10^−5^) ^c^	0.8510 ^c^
77	7.86	C_34_H_46_O_15_	695.2499	0.6	693.2490	−0.7	694.2495	Nomilin glycoside	2.10 (0.012) ^a^, 1.42 (0.0010) ^b^, 1.44 (0.0090) ^c^	-
78 *	8.38	C_28_H_34_O_9_	515.2447	−0.2	513.2444	−0.8	514.2448	Nomilin	6.40 (0.0011) ^b^, 4.05 (0.0063) ^c^	-
Abscisic acid										
79 *	4.14	C_15_H_20_O_4_	265.1380	2.3	263.1373	−0.4	264.1374	Abscisic acid	1.62 (3.95 × 10^−5^)a, 5.74 (0.035) ^b^, 7.54 (1.72 × 10^−5^) ^c^	-

* metabolites were confirmed by authentic standards; ^a^ group of QP/CP; ^b^ group of ZS/ZQ; ^c^ group of species (CA/CR).

**Table 3:** Origins of the collected 37 QP, CP, ZS and ZQ samples.

No.	Original Plant	Local Name	Location and Collection Time	Growing Environment
Pericarps of the ripe fruit of *Citrus reticulate* Blanco				
1	*Citrus reticulate* Blanco	Chenpi	Fengjie, Sichuan; August 2015	Field margins (29° N 106° E; Alt.200–230 m)
2	*Citrus reticulate* Blanco	Chenpi	Qinglong, Guizhou; September 2015	Hillsides (25° N 105° E; Alt.1200–1301 m)
3	*Citrus reticulate* Blanco	Chenpi	Jiangjin, Sichuan; September 2014	Hillsides (29° N 106° E; Alt.231 m)
4	*Citrus reticulate* Blanco	Chenpi	Jiangjin, Sichuan; October 2014	Field margins (29° N 106° E; Alt.200–230 m)
5	*Citrus reticulate* Blanco	Chenpi	Ganzhou, Jiangxi; September 2015	Plain (26° N 115° E; Alt.500 m)
6	*Citrus reticulate* Blanco	Chenpi	Ganzhou, Jiangxi; September 2015	Plain (25° N 115° E; Alt.520 m)
7	*Citrus reticulate* Blanco	Chenpi	Qinglong, Guizhou; October 2015	Hillsides (25° N 105° E; Alt.1200–1300 m)
8	*Citrus reticulate* Blanco	Chenpi	Huangyan, Zhejiang; September 2013	Field margins (28° N 121° E; Alt.45m)
9	*Citrus reticulate* Blanco	Chenpi	Jiangjin, Sichuan; August 2015	Hillsides (29° N 106° E; Alt.238 m)
Pericarps of the young or immature fruit of *Citrus reticulate* Blanco				
10	*Citrus reticulate* Blanco	Qingpi	Xingan, Jiangxi; May 2015	Hillsides (27° N 115° E; Alt.50–60 m)
11	*Citrus reticulate* Blanco	Qingpi	Jiangjin, Sichuan; May 2015	Hillsides (29° N 106° E; Alt.220 m)
12	*Citrus reticulate* Blanco	Qingpi	Xingan, Jiangxi; June 2015	Hillsides (27° N 115° E; Alt.50–60 m)
13	*Citrus reticulate* Blanco	Qingpi	Xingan, Jiangxi; May 2014	Hillsides (27° N 115° E; Alt.50–60 m)
14	*Citrus reticulate* Blanco	Qingpi	Jiangjin, Sichuan; May 2014	Hillsides (29° N 106° E; Alt.220 m)
15	*Citrus reticulate* Blanco	Qingpi	Xingan, Jiangxi; June 2014	Hillsides (27° N 115° E; Alt.50–60 m)
16	*Citrus reticulate* Blanco	Qingpi	Xingan, Jiangxi; May 2013	Hillsides (27° N 115° E; Alt.50–60 m)
17	*Citrus reticulate* Blanco	Qingpi	Jiangjin, Sichuan; May 2013	Hillsides (29° N 106° E; Alt.220 m)
18	*Citrus reticulate* Blanco	Qingpi	Xingan, Jiangxi; June 2013	Hillsides (27° N 115° E; Alt.50–60 m)
19	*Citrus reticulate* Blanco	Qingpi	Ganzhou, Jiangxi; June 2015	Hillsides (27° N 115° E; Alt.50–60 m)
Immature fruit of *Citrus aurantium* L.				
20	*Citrus aurantium* cv Xiucheng	Xiucheng	Xingan, Jiangxi; July 2014	Plain (27° N 115° E; Alt.20–30 m)
21	*Citrus aurantium* L.	Sour orange	Ezhou, Hubei; July 2013	Hillsides (21° N 110° E; Alt.300 m)
22	*Citrus aurantium* cv Xiucheng	Xiucheng	Xingan, Jiangxi; July 2015	Plain (27° N 115° E; Alt.20–30 m)
23	*Citrus aurantium* L.	Jiangjin sour orange	Jiangjin, Sichuan; July 2014	Hillsides (29° N 106° E; Alt.231 m)
24	*Citrus aurantium* cv Jizicheng	Jizhicheng	Zhangshu, Jiangxi; July 2015	Plain (27° N 115° E; Alt.20–30 m)
25	*Citrus aurantium* L.	Sour orange	Jian, Jiangxi; July 2015	Plain (27° N 115° E; Alt.20–30 m)
26	*Citrus aurantium* L.	Sour orange	Zhangshu, Jiangxi; July 2014	Plain (27° N 115° E; Alt.20–30 m)
27	*Citrus aurantium* L.	Sour orange	Jian, Jiangxi; July 2014	Plain (27°N115°E; Alt.20–30 m)
28	*Citrus aurantium* × P. trifoliata	Citrange	Yuanjiang, Hunan; July 2014	Hillsides (29° N 112° E; Alt.310 m)
Young fruit of *Citrus aurantium* L.				
29	*Citrus aurantium* cv Xiucheng	Xiucheng	Xingan, Jiangxi; May 2014	Plain (27° N 115° E; Alt.20–30 m)
30	*Citrus aurantium* L.	Sour orange	Jiangjin, Sichuan; June 2014	Field margins (29° N 106° E; Alt.200–230 m)
31	*Citrus aurantium* cv Xiucheng	Xiucheng	Xingan, Jiangxi; May 2015	Plain (27° N 115° E; Alt.20–30 m)
32	*Citrus aurantium* L.	Jiangjin sour orange	Jiangjin, Sichuan; June 2014	Hillsides (29° N 106° E; Alt.231 m)
33	*Citrus aurantium* cv Daidai	Daidai	Jiangjin, Sichuan; May 2014	Field margins (29° N 106° E; Alt.200–230 m)
34	*Citrus aurantium* cv Xiucheng	Xiucheng	Zhangshu, Jiangxi; June 2014	Plain (27° N 115° E; Alt.20–30 m)
35	*Citrus aurantium* cv Morocco sour orange	Morocco sour orange	Huangyan, Zhejiang; May 2013	Field margins (28° N 121° E; Alt.45 m)
36	*Citrus aurantium* L.	Xiucheng	Qingjiang, Jiangxi; June 2012	Plain (27° N 114° E; Alt.100–200 m)
37	*Citrus aurantium* cv Xiucheng	Xiucheng	Xingan, Jiangxi; June 2015	Plain (27° N 115° E; Alt.20–30 m)

## Supplementary Materials

The supplementary materials are available online.
