# Supplementary material for: Chemotaxonomic Classification Applied to the Identification of Two Closely-Related Citrus TCMs Using UPLC-Q-TOF-MS-Based Metabolomics
Source: Molecules. 2017 Oct 13;22(10):1721. doi: 10.3390/molecules22101721 (PMC6151587; doi:10.3390/molecules22101721)

Contents:

The explanation for the question of inequivalent tissues/organs and inequivalent developmental stages between the species.

Table S1. The precision, repeatability and stability of UPLC-Q-TOF/MS with the ACQUITY UPLC HSS T3 column.

Table S2. The precision, repeatability and stability of UPLC-Q-TOF/MS with the ACQUITY UPLC BEH HILIC column.

Table S3. The rest of the differential primary metabolites between the *Citrus reticulate* Blanco and *Citrus aurantium* L. group profiles.

Table S4. The levels and factors investigated in the PB test.

Table S5. The weight of pulps and fruits of Zhishi and Zhiqiao.

Figure S1. Diagnostic efficacy evaluation using ROC curves of altered primary metabolites.

Figure S2. Diagnostic efficacy evaluation using the ROC curves of altered metabolites between different groups detected by UPLC-Q-TOF/MS. The optimal cutoffs using the closest to top-left corner and the area under ROC curves with a 95% confidence interval are displayed (AUC > 0.85). (A) The ROC curves of the QP/CP group, ROC curve-based model evaluation (AUC = 0.934). (B) The ROC curves of ZS/ZQ group, ROC curve-based model evaluation (AUC = 1.000). (C) The ROC curves of the CR/CA group, ROC curve-based model evaluation (AUC = 0.984).

Figure S3. The main effect plots of different compounds. The PB test was performed to investigate which factors significantly affected the different markers. The two conditions that we discuss below were assessed, including species and ripening stages. Main effects plots were used to examine differences between mean levels for one or more factors. In the main effect plots, when the line is horizontal (parallel to the x-axis), there is no main effect. Each level of the factor affects the response in the same way, and the response mean is the same across all factor levels. When the line is not horizontal, then there is a main effect. Different levels of the factor affect the response differently. The red line in the figure means the influence of species; the blue line represents the differences of QP/CP group; the orange line is for the ZS/ZQ group; and the green line means the discriminations in both the QP/CP group and the ZS/ZQ group.

Figure S4. Images of dried voucher specimens.

Figure S5. The pulps and peels of Zhishi and Zhiqiao samples. (A1) The pulps of Zhiqiao; (A2) the peels of Zhiqiao; (B1) the pulps of Zhishi; (B2) the peels of Zhishi.

Figure S6. The BPI chromatograms of Zhishi and Zhiqiao samples. (A1) Pulp samples of ZQ. (A2) Peel samples of ZQ. (A3) Fruit samples of ZQ. (B1) Pulp samples of ZS. (B2) Peels sample of ZS. (B3) Fruit samples of ZS.

Figure S7. The PCA and PLS-DA plot of Qingpi and Zhiqiao. (A) The PCA plot of Qingpi and Zhiqiao. (B) The PLS-DA plot of Qingpi and Zhiqiao.

Figure S8. The BPI chromatograms of the four *Citrus* samples by the ACQUITY UPLC HSS T3 column. (A) Zhishi; (B); Zhiqiao; (C) Chenpi; (D) Qingpi.

Figure S9. The BPI chromatograms of the four *Citrus* samples by the ACQUITY UPLC BEH HILIC column. (A) Zhishi; (B) Zhiqiao; (C) Chenpi; (D) Qingpi.

Figure S10. The PCA score plot and PLS-DA score plot of the peels and fruits. (A) The PCA score plot. (B) The PLS-DA score plot. The samples of peels are marked in green; the samples of fruits are marked in blue.

The Explanation for the Question of Inequivalent Tissues/Organs and Inequivalent Developmental Stages between the Species

The explanation for the question of inequivalent tissues/organs:

The four samples selected in this research are Zhishi (ZS, young fruit of *Citrus aurantium* L.), Zhiqiao (ZQ, immature fruit of *Citrus aurantium* L.), Qingpi (QP, peels of the immature fruit of *Citrus reticulate* Blanco) and Chenpi (CP, peels of the ripe fruit of *Citrus reticulate* Blanco). It seems tissues of the different species are inequivalent. However, *Citrus aurantium* L. is one of the special species in *Citrus* with small fruits, thin pulps and thick peels, which can only be used as decoration and for phytotherapy. As shown in Figure S4, Zhishi and Zhiqiao are the young and immature fruits of *Citrus aurantium* L., which have thinner pulps and thicker peels. Compared with Zhiqiao, Zhishi is constituted by less pulp, as it is the young fruit of *Citrus aurantium* L.

Moreover, in order to compare the differences of fruits and peels in different *Citrus* species, the pulps of the samples Zhishi and Zhiqiao were scooped, and the remaining tissues were equivalent to Qingpi and Chenpi. Meanwhile, the comparison of pulps and peels of the Zhishi and Zhiqiao samples is shown in Figure S5.

The weight of the pulps and fruits of Zhishi and Zhiqiao samples are listed in Table S5, and the weight rate of pulps and fruits was calculated by the following equation:

$$\mathbf{Rate} = \frac{\boldsymbol{W_{pulps}}}{\boldsymbol{W_{fruits}}} \times \mathbf{100\%}$$

The results indicated that the rate of pulps of Zhiqiao was lower than 6.8%, whereas the rate of Zhishi was lower than 3.5%. The average of the rate was 6.0% and 3.1%, respectively.

The established UPLC-Q-TOF-MS method was employed for the determination of the metabolite profiles of pulps, peels and fruits in the Zhishi and Zhiqiao samples. All the samples were pulverized and sifted through a 60-mesh sieve. For the ACQUITY UPLC HSS T3 column, the samples were weighed powder (0.5 g) placed in a conical flask and then sonicated three times with 5 mL of 50% MeOH for 30 min. The supernatant solution was combined and filtered through a 0.22-μm filter membrane. Additionally, for the ACQUITY UPLC BEH HILIC column, the dried powder (0.5 g) was weighed accurately into a 5-mL conical flask with a stopper, and 10 mL water were added accurately. After weighing the filled flask accurately, ultrasonication (40 kHz) was carried out at room temperature for 30 min, then weighed again, and the same solvent (water) was

added to compensate for the lost weight during the extraction as needed. The supernatant solution was combined and filtered through a 0.22-μm filter membrane, as well. The conditions of chromatographic spectrometry and mass spectrometry were the same as Sections 4.3 and 4.4 in the manuscript. The BPI chromatograms of peels, pulps and fruits in each of the ZS and ZQ samples are shown in Figure S6.

According to the above experiments, there were no significant differences between peels and fruits. As reported in the literatures many research works focus on the peel of *Citrus* species for it possesses various well-known compounds and biological activities, including anti-inflammatory, antioxidant, antitumor, antimicrobial and anti-atherosclerosis activities. (Duan et al., 2017; Jung et al., 2017; Xi et al., 2017)

The explanation for the question of inequivalent developmental stages between the species: Based on the above comments, the samples of Qingpi (peels of the young or immature fruit of *Citrus reticulate* Blanco) and Zhiqiao (immature fruit of *Citrus aurantium* L.; the constituents in fruit and peels were similar) were harvested in the same period. In fact, Qingpi and Zhiqiao were in the equivalent developmental stages between the different species and could be clearly separated, according to the PCA and OPLS-DA plot shown in Figure S6. Moreover, the comparison we performed between the different developmental stages, such as Zhishi/Zhiqiao and Qingpi/Chenpi, was in the same species.

References:

Duan, L., Dou, L. L., Yu, K. Y., Guo, L., Bai-Zhong, C., Li, P., Liu, E. H., 2017. Polymethoxyflavones in peel of Citrus reticulata 'Chachi' and their biological activities. Food Chem 234, 254-261.

Jung, J., Friedrich, L. M., Danyluk, M. D., Schaffner, D. W., 2017. Quantification of Transfer of Salmonella from Citrus Fruits to Peel, Edible Portion, and Gloved Hands during Hand Peeling. J Food Prot 80, 933-939.

Xi, W., Lu, J., Qun, J., Jiao, B., 2017. Characterization of phenolic profile and antioxidant capacity of different fruit part from lemon (Citrus limon Burm.) cultivars. J Food Sci Technol 54, 1108-1118.

Table S1. The precision, repeatability and stability of UPLC-Q-TOF/MS with the ACQUITY UPLC HSS T3 column.

Compound	Precision (RSD, %)		Repeatability	Stability (24 h)
	intra-day	inter-day	(RSD, %)	(RSD, %)
7-hydroxycoumarin	2.28	2.44	1.52	1.99
bergapten	0.36	2.70	0.85	0.32
narirutin	1.35	2.81	0.69	0.95
hesperidin	0.83	2.46	1.07	1.15
naringin	2.04	2.74	1.37	1.06
apigetrin	2.29	2.01	2.40	2.51
kaempferitrin	2.54	3.19	2.46	2.26
diosmetin-7-O-glucoside	1.88	2.62	2.66	1.67
sinensetin	2.26	2.74	1.22	2.17
eriocitrin	1.92	2.79	2.23	2.66
neohesperidin	2.53	1.93	1.91	1.86
naringenin	1.80	3.26	2.70	1.80
hesperetin	2.28	1.45	2.36	2.76
luteolin	1.44	2.00	1.52	1.31
rutin	2.36	1.97	2.07	2.18
quercetin	1.60	2.65	1.67	1.85
tangeretin	1.26	2.16	2.79	2.03
5-femethylnobiletin	1.45	2.02	1.35	2.24
nobiletin	0.80	2.63	1.46	1.61
auraptene	2.92	3.36	2.30	2.96
bergamottin	2.18	1.99	2.12	6.13
rhoifolin	2.43	2.22	1.12	2.88
apigenin	1.08	1.13	1.13	0.89
scoparone	2.76	0.82	0.87	1.78
isorhamnetin-3-O-glucoside	0.90	3.31	2.66	3.11
diosmetin	0.81	1.18	0.78	4.15
poncirin	1.31	2.21	1.21	2.78
eriodictyol	1.22	3.31	2.11	0.98
xanthotoxol	1.10	2.66	0.99	3.19
acacetin	0.98	1.77	1.87	2.77
isosakuranetin	0.78	2.34	1.44	2.67
imperatorin	1.22	2.10	2.19	1.87
limonin	1.32	1.89	0.76	2.15
nomilin	1.09	2.76	2.11	2.10
abscisic acid	2.11	3.34	0.99	1.21

Table S2. The precision, repeatability and stability of UPLC-Q-TOF/MS with the ACQUITY UPLC BEH HILIC column.

Compound	Precision (RSD, %)		Repeatability	Stability (24 h)
	intra-day	inter-day	(RSD, %)	(RSD, %)
shikimic acid	1.18	1.92	1.44	0.89
aspartic acid	2.09	0.99	1.23	1.14
asparagine	1.77	2.13	0.76	0.65
theanine	0.98	1.88	1.03	1.43
glutamic acid	1.23	2.74	1.56	1.22
glutamine	1.86	2.11	0.89	1.20
quinic acid	1.72	1.34	1.43	0.77
p-coumaric acid	1.23	0.98	1.42	1.13
UMP	2.11	1.17	1.12	1.21

Table S3. The rest of the differential primary metabolites between the *Citrus reticulate* Blanco and *Citrus aurantium* L. group profiles.

No.	ESI mode	Rt_Mass	Identification	*p* Value	AUC
M49	+	355.2692_11.47	MG(18:2/0:0/0:0)	$5.88E^{-4}$	0.8450
M50	+	517.3246_9.32	PC(18:3/0:0)	0.0134	0.8392
M51	-	513.1253_3.52	PE(2:0/0:0)	0.0029	0.8363
M52	+	227.1263_3.46	MG(7:0/0:0/0:0)	0.0017	0.8333
M53	+	515.2467_9.18	PC(18:4/0:0)	0.011	0.8216
M54	-	478.2931_10.28	PE(18:1/0:0)/PE(P-16:0/2:0)	0.1949	0.8129
M55	-	812.5453_9.69	GPSer(18:2/20:1)/GPSer(20:1/18:2)	0.0024	0.8099
M56	-	885.3055_4.07	GPSer(2:0/7:0)/GPSer(7:0/2:0)	0.1698	0.8072
M57	+	405.1187_7.84	Cassiaside	0.0011	0.8012
M58	+	289.1256_2.83	Duloxetine	0.0010	0.7982
M59	+	397.1976_3.08	GPA(10:0/4:0)/GPA(4:0/10:0)	0.0071	0.7807
M60	+	379.2849_9.21	MG(20:4/0:0/0:0)	0.0090	0.7748
M61	+	163.0755_3.84	3,6-Dimethyl-2(3H)-benzofuranone	0.0018	0.7748
M62	-	597.2175_3.20	PE(5:0/5:0)	0.032	0.7651
M63	-	713.1567_3.22	GPSer(2:0/3:0)/GPSer(3:0/2:0)	0.021	0.7651
M64	+	180.1024_4.38	3,5-Dimethylphenyl methylcarbamate	0.0089	0.7532
M65	+	313.1076_8.13	GPA(2:0/6:0)/GPA(6:0/2:0)	0.031	0.7488
M66	+	411.2168_4.74	GPA(10:0/3:0)/GPA(3:0/10:0)	0.0045	0.7453
M67	+	351.2506_4.32	MG(16:3/0:0/0:0)	0.056	0.7392
M68	-	681.2395_3.25	GPEtn(2:0/5:0)/GPEtn(5:0/2:0)	0.022	0.7181
M69	-	685.2713_3.55	PE(6:0/6:0)	0.019	0.7091
M70	+	299.0892_6.52	GPA(2:0/6:0)/GPA(6:0/2:0)	0.078	0.7067
M71	+	369.1681_8.73	GPA(15:0/2:0)/GPA(2:0/15:0)	0.1925	0.7056
M72	+	383.1868_7.82	GPA(10:0/3:0)/GPA(3:0/10:0)	0.1848	0.6981

M73	-	825.3530_6.41	GPEtn(2:0/8:0)/GPEtn(8:0/2:0)	0.0981	0.6981
M74	-	991.5516_7.11	GPA(10:0/9:0)/GPA(9:0/10:0)	0.1343	0.6848
M75	-	651.4108_10.03	MGDG(18:4/9:0)	0.1678	0.6721
M76	-	735.5053_10.15	MGDG(15:1/18:3)	0.088	0.6721
M77	-	629.2082_3.82	PE(4:0/0:0)	0.2467	0.6672
M78	-	505.3014_10.78	MGDG(10:0/6:0)/MGDG(6:0/10:0)	0.3022	0.6520
M79	-	611.2833_7.97	GPIns(14:1/4:0)	0.1898	0.6473
M80	-	1107.5977_6.25	GPA(20:5/6:0)/GPA(6:0/20:5)	0.2467	0.6288
M81	+	180.1022_2.79	2,3-Dihydro-5-(3-hydroxypropanoyl)-1H-pyrrolizine	0.3452	0.6170
M82	+	476.2776_9.21	PE(18:3/0:0)	0.4671	0.6140
M83	+	297.1547_2.99	MG(3:0/0:0/0:0)	0.1284	0.6111
M84	-	1045.5748_4.34	GPEtn(14:0/4:0)/GPEtn(4:0/14:0)	0.097	0.6111
M85	+	180.1022_2.83	3,4-Methylenedioxyamphetamine	0.4364	0.6081
M86	-	709.2694_4.76	GPEtn(2:0/6:0)/GPEtn(6:0/2:0)	0.2096	0.5467
M87	-	597.2951_8.94	SQDG(10:0/8:0)/SQDG(8:0/10:0)	0.7326	0.5117
M88	+	478.2938_9.70	PE(18:2/0:0)	0.6474	0.5029

Table S4. The levels and factors investigated in the Plackett–Burman (PB) test.

	Factors	−1 level	+1 level
A	species	*Citrus reticulate* Blanco	*Citrus aurantium* L.
B	ripening stages	young	mature

Table S5. The weight of pulps and fruits of Zhishi and Zhiqiao.

No.	Weight of pulps (g)	Weight of fruits (g)	Rate (%)
Immature fruit of *Citrus aurantium* L. (Zhiqiao)			
21	1.58	30.96	5.1
25	1.51	29.09	5.2
26	2.08	32.11	6.5
27	2.06	30.28	6.8
28	2.11	32.93	6.4
Average			6.0
Young fruit of *Citrus aurantium* L. (Zhishi)			
30	0.23	7.81	3.3
32	0.24	8.17	2.9
33	0.23	8.32	2.8
34	0.22	7.34	3.1
36	0.27	7.71	3.5
Average			3.1

Figure S1. Diagnostic efficacy evaluation using the ROC curves of altered primary metabolites.

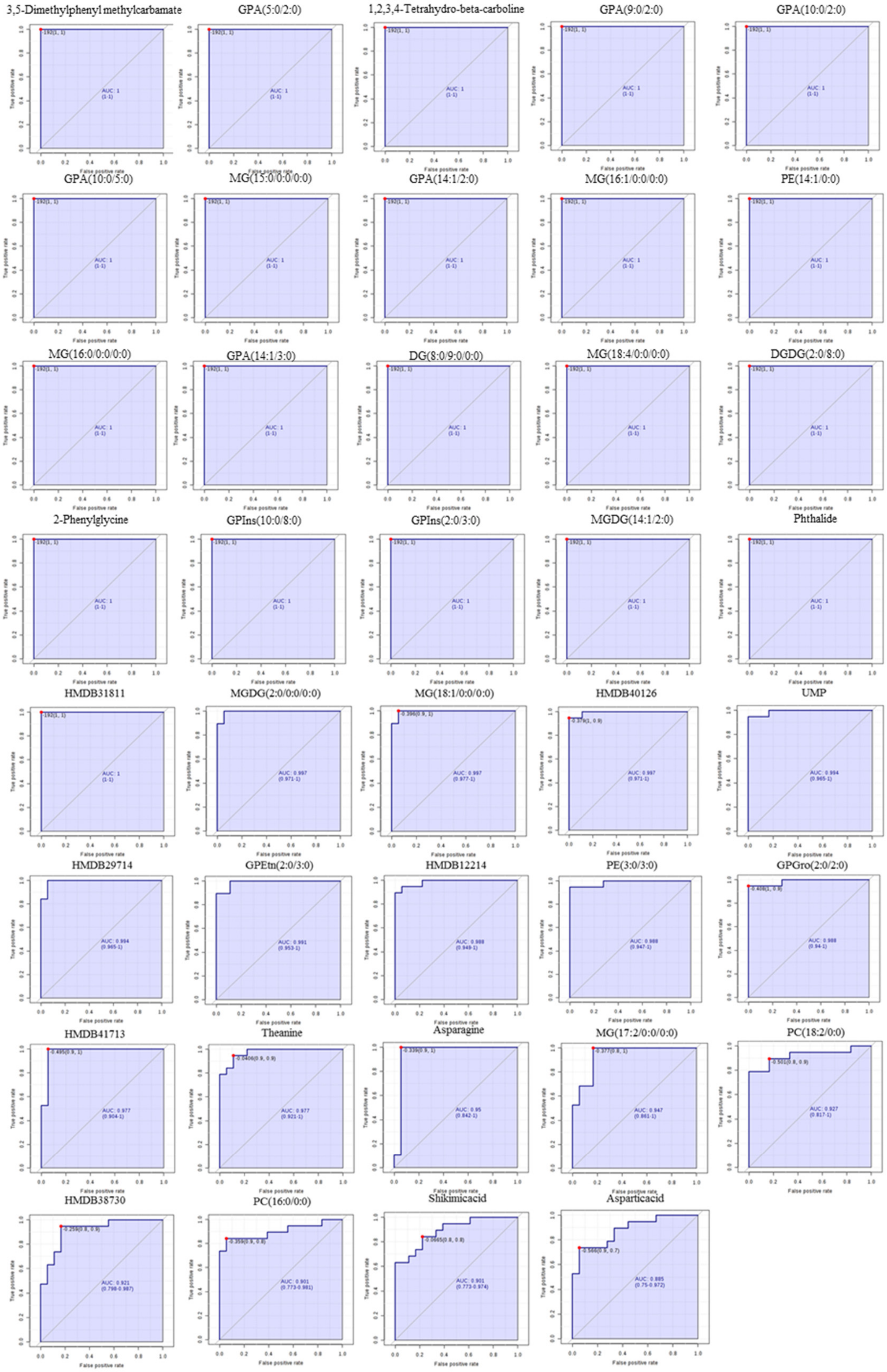

Figure S2. Diagnostic efficacy evaluation using the ROC curves of altered metabolites between different groups detected by UPLC-Q-TOF/MS. The optimal cutoffs using the closest to top-left corner and the area under ROC curves with a 95% confidence interval are displayed (AUC > 0.85). (**A**) The ROC curves of the QP/CP group, ROC curve-based model evaluation (AUC = 0.934). (**B**) The ROC curves of the ZS/ZQ group, ROC curve-based model evaluation (AUC = 1.000). (**C**) The ROC curves of the CR/CA group, ROC curve-based model evaluation (AUC = 0.984).

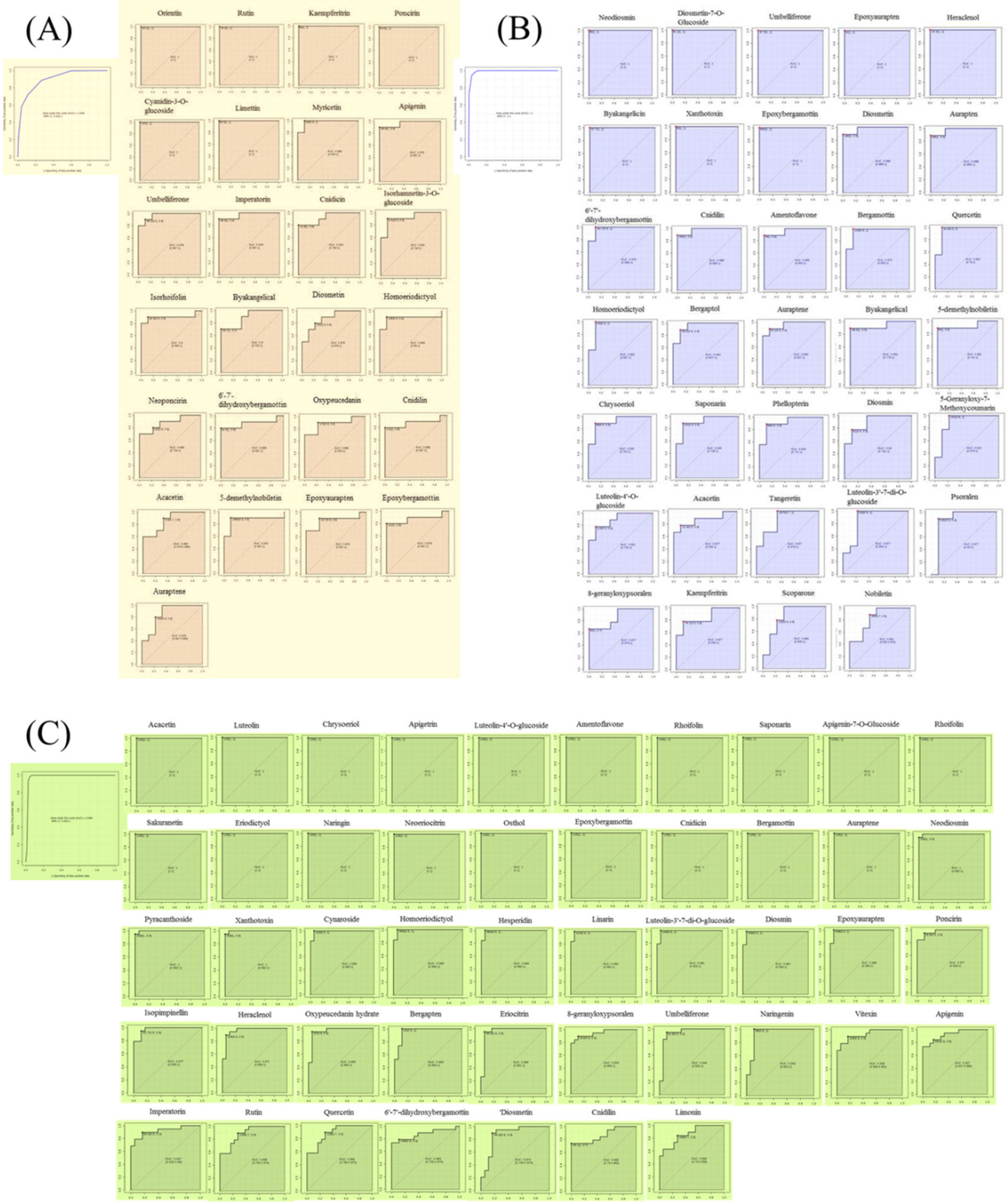

Figure S3. The main effect plots of different compounds. The PB test was performed to investigate which factors significantly affected the different markers. The two conditions that we discuss below were assessed, including species and ripening stages. Main effects plots were used to examine differences between mean levels for one or more factors. In the main effect plots, when the line is horizontal (parallel to the x-axis), there is no main effect. Each level of the factor affects the response in the same way, and the response mean is the same across all factor levels. When the line is not horizontal, then there is a main effect. Different levels of the factor affect the response differently. The red line in the figure means the influence of species; the blue line represents the differences of QP/CP group; the orange line is for the ZS/ZQ group; and the green line means the discriminations in both the QP/CP group and the ZS/ZQ group.

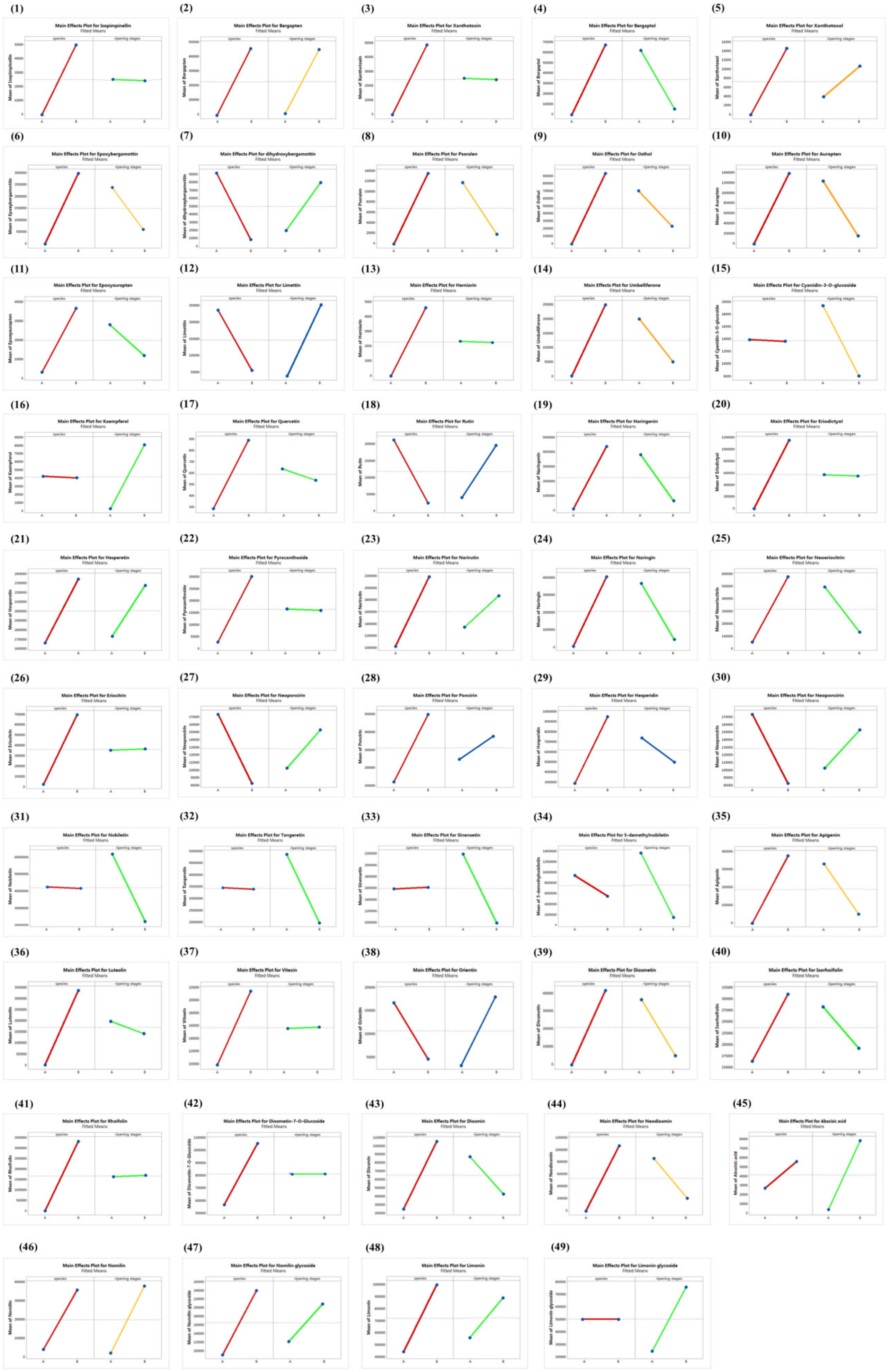
(1)
Main Effects Plot for Isopimpinellin
(2)
Main Effects Plot for Bergapten
(3)
Main Effects Plot for Xanthotoxin
(4)
Main Effects Plot for Bergaptol
(5)
Main Effects Plot for Xanthotoxol
(6)
Main Effects Plot for Epoxybergamottin
(7)
Main Effects Plot for dihydroxybergamottin
(8)
Main Effects Plot for Psoralen
(9)
Main Effects Plot for Osthol
(10)
Main Effects Plot for Aurapten
(11)
Main Effects Plot for Epoxyaurapten
(12)
Main Effects Plot for Limettin
(13)
Main Effects Plot for Herniarin
(14)
Main Effects Plot for Umbelliferone
(15)
Main Effects Plot for Cyanidin-3-O-glucoside
(16)
Main Effects Plot for Kaempferol
(17)
Main Effects Plot for Quercetin
(18)
Main Effects Plot for Rutin
(19)
Main Effects Plot for Naringenin
(20)
Main Effects Plot for Eriodictyol
(21)
Main Effects Plot for Hesperetin
(22)
Main Effects Plot for Pyrocanthoside
(23)
Main Effects Plot for Narirutin
(24)
Main Effects Plot for Naringin
(25)
Main Effects Plot for Neoeriocitrin
(26)
Main Effects Plot for Eriocitrin
(27)
Main Effects Plot for Neoponcirin
(28)
Main Effects Plot for Poncirin
(29)
Main Effects Plot for Hesperidin
(30)
Main Effects Plot for Neoponcirin
(31)
Main Effects Plot for Nobiletin
(32)
Main Effects Plot for Tangeretin
(33)
Main Effects Plot for Sinensetin
(34)
Main Effects Plot for 5-demethylnobiletin
(35)
Main Effects Plot for Apigenin
(36)
Main Effects Plot for Luteolin
(37)
Main Effects Plot for Vitexin
(38)
Main Effects Plot for Orientin
(39)
Main Effects Plot for Diosmetin
(40)
Main Effects Plot for Isorhoifolin
(41)
Main Effects Plot for Rhoifolin
(42)
Main Effects Plot for Diosmetin-7-O-Glucoside
(43)
Main Effects Plot for Diosmin
(44)
Main Effects Plot for Neodiosmin
(45)
Main Effects Plot for Abscisic acid
(46)
Main Effects Plot for Nomilin
(47)
Main Effects Plot for Nomilin glycoside
(48)
Main Effects Plot for Limonin
(49)
Main Effects Plot for Limonin glycoside

Figure S4. Images of dried voucher specimens.

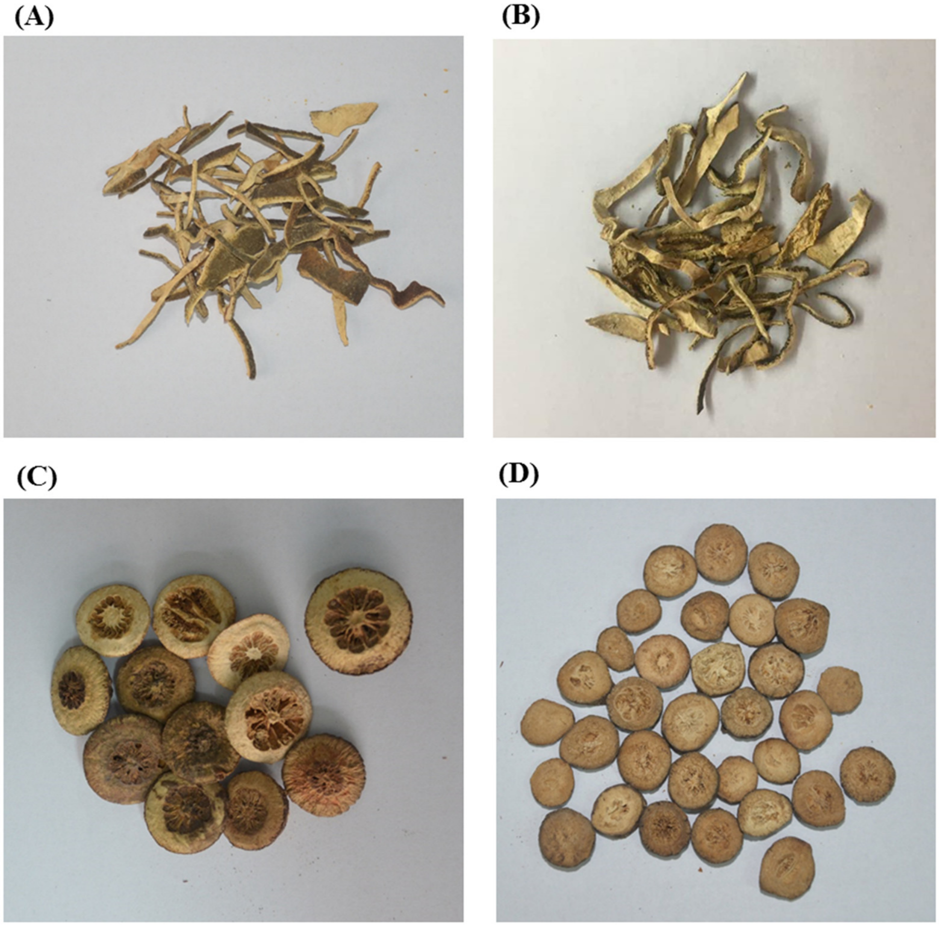

Figure S5. The pulps and peels of the Zhishi and Zhiqiao samples. (**A1**) The pulps of Zhiqiao; (**A2**) the peels of Zhiqiao; (**B1**) the pulps of Zhishi; (**B2**) the peels of Zhishi.

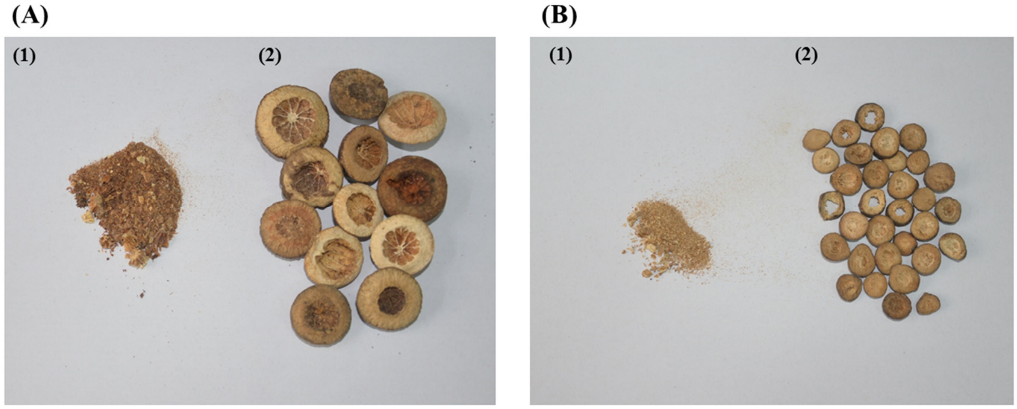

Figure S6. The BPI chromatograms of the Zhishi and Zhiqiao samples. (**A1**) Pulp samples of ZQ. (**A2**) Peels sample of ZQ. (**A3**) Fruit samples of ZQ. (**B1**) Pulp samples of ZS. (**B2**) Peel samples of ZS. (**B3**) Fruit samples of ZS.

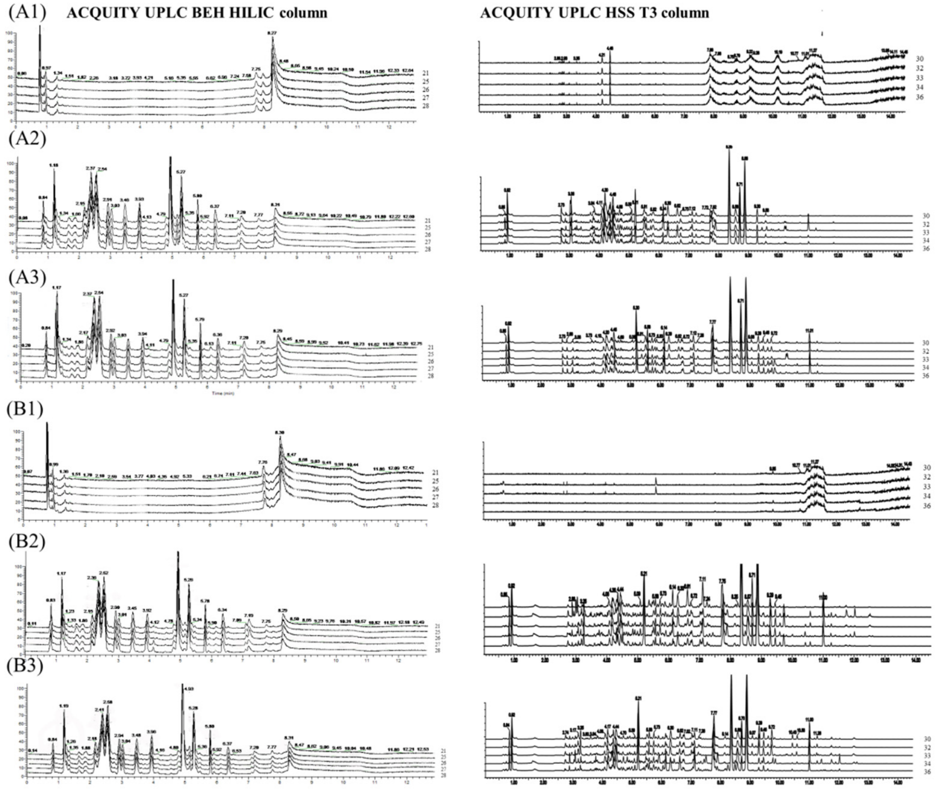

Figure S7. The PCA and PLS-DA plot of Qingpi and Zhiqiao. (**A**) The PCA plot of Qingpi and Zhiqiao. (**B**) The PLS-DA plot of Qingpi and Zhiqiao.

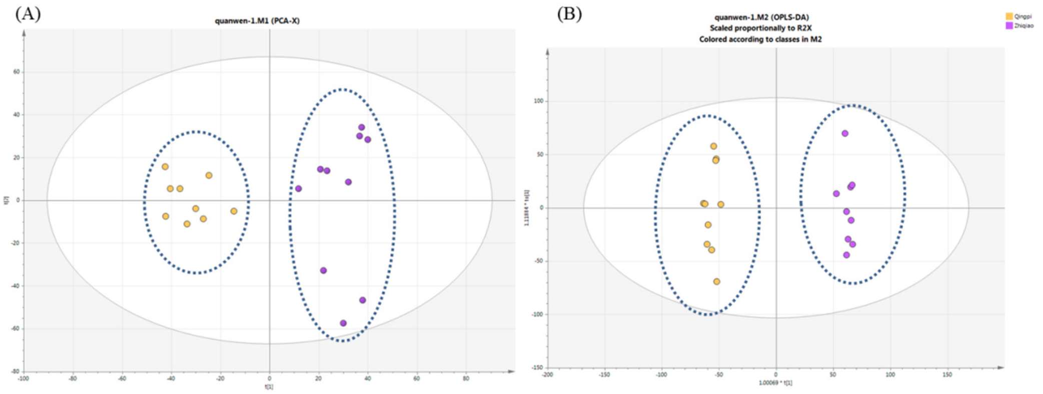

Figure S8. The BPI chromatograms of the four *Citrus* samples by the ACQUITY UPLC HSS T3 column. (**A**) Zhishi; (**B**) Zhiqiao; (**C**) Chenpi; (**D**) Qingpi.

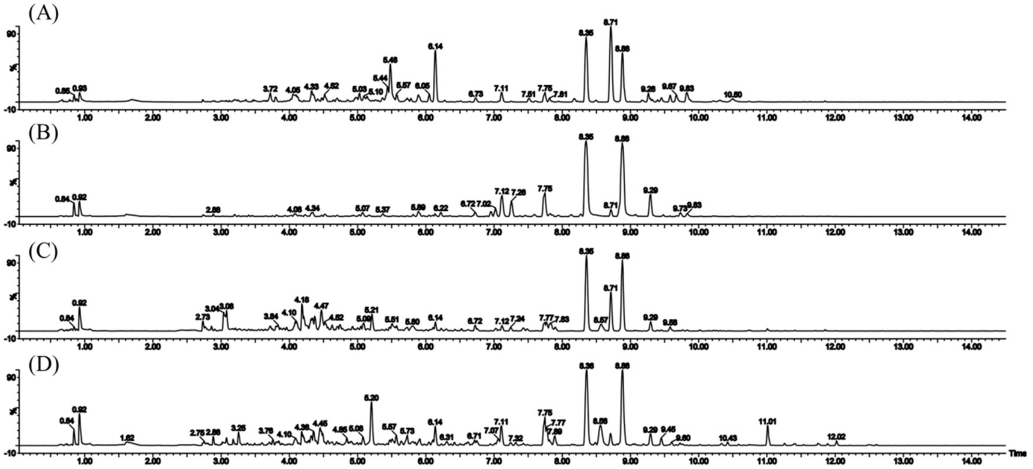

Figure S9. The BPI chromatograms of the four *Citrus* samples by the ACQUITY UPLC BEH HILIC column. (**A**) Zhishi; (**B**) Zhiqiao; (**C**) Chenpi; (**D**) Qingpi.

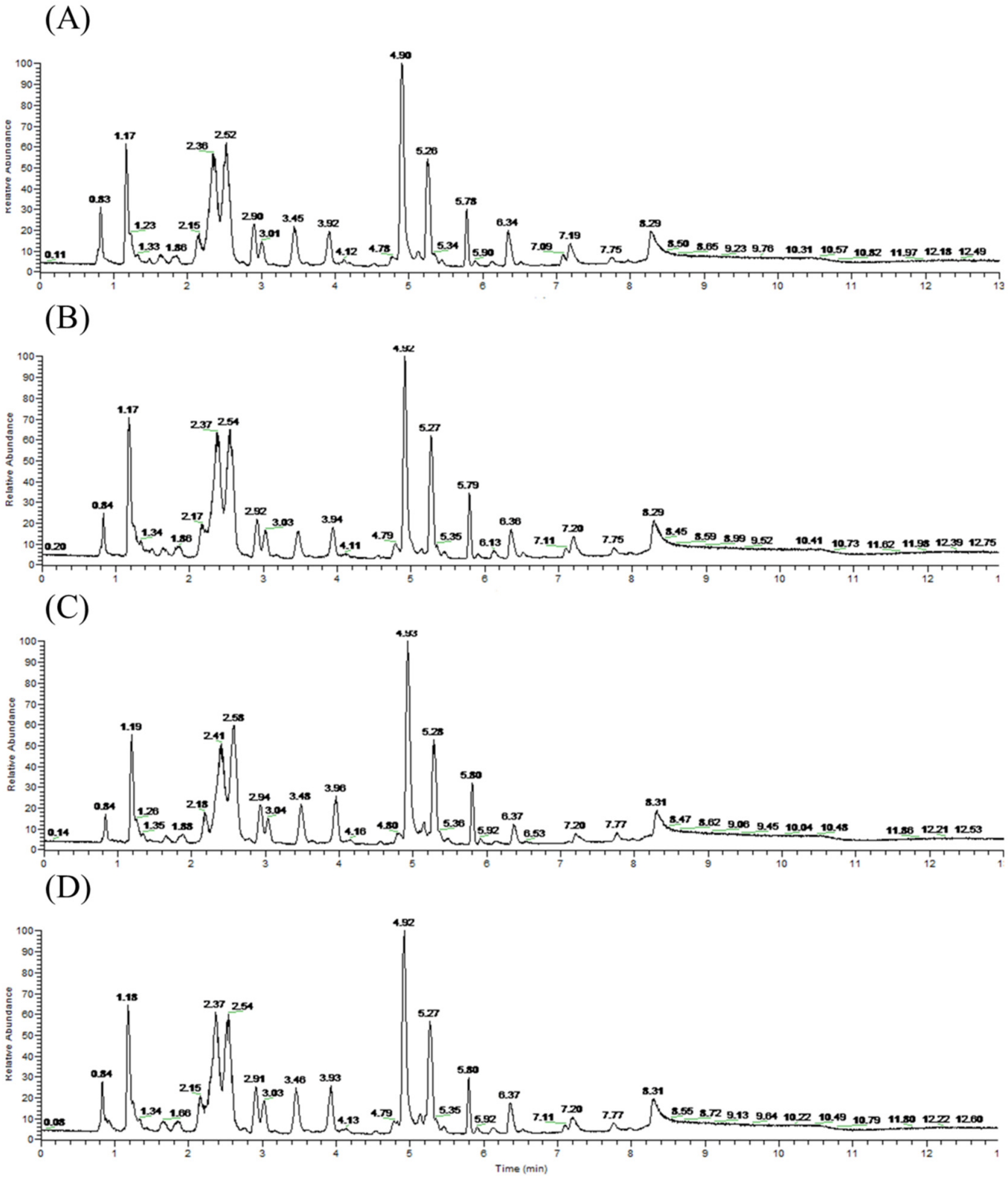

Figure S10. The PCA score plot and PLS-DA score plot of the peels and fruits. (**A**) The PCA score plot. (**B**) The PLS-DA score plot. The samples of peels are marked in green; the samples of fruits are marked in blue.

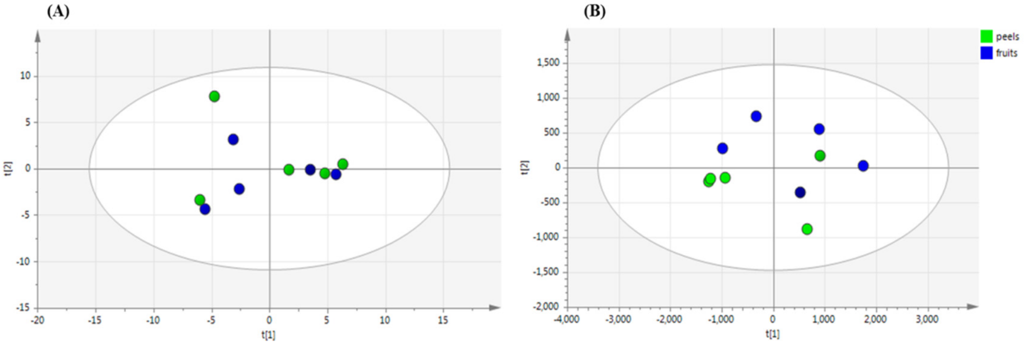

Supplement: Supplementary file 1 [file molecules-22-01721-s001.pdf]
